# Population Genetics of the Greater Grison (*Galictis vittata*; Mustelidae, Carnivora) and How Changes Since Miocene–Pliocene Have Shaped the Phylogenetic Relationships Between Grison Species and Other Neotropical Mustelid Genera

**DOI:** 10.3390/ani16152364

**Published:** 2026-08-02

**Authors:** Manuel Ruiz-García, Juan Esteban Fula-Rodríguez

**Affiliations:** Laboratorio de Genética de Poblaciones Molecular-Biología Evolutiva, Unidad de Genética, Departamento de Biología, Facultad de Ciencias, Pontificia Universidad Javeriana, Bogotá 110311, Colombia; juane.fula@javeriana.edu.co

**Keywords:** demographic population changes, *Galictis cuja*, *Galictis vittata*, genetic diversity, genetic heterogeneity, Ictonychinae, *Lyncodon patagonicus*, Lyncodontini, miocene, mitochondrial DNA, neotropics, pliocene, phylogenies, population genetics

## Abstract

Mustelids are a very diverse carnivoran group, boasting the largest number of carnivoran species today, with the most extreme ecological adaptations. Nevertheless, Neotropical Lyncodontini, one Neotropical mustelid subfamily, is one of the least studied from a genetic and evolutionary perspective. The use of small fragments of three mitochondrial (mt) markers (*NADH5*, *12S rRNA*, and *D-loop*) proved to be very useful for recovering relevant information on the evolutionary history of Neotropical Lyncodontini species of the genera *Galictis* and *Lyncodon*. This study used the largest population sample analyzed to date for *G. vittata* (13 specimens from Guatemala, Colombia, Ecuador, Peru, Bolivia, and French Guiana) and reports for the first time sequences for three mt markers for *L. patagonicus* other than the mt*Cyt-b* gene. The main results obtained were the following. (1) The haplotype and nucleotide diversity of *G. vittata* was lower than these statistics measured in other studies of *G. cuja* in southern and southeastern Brazil. (2) There was evidence from two analyses of a possible population expansion of the female lineages of *G. vittata*, which began between 635,000 and 370,000 years ago (ya) (mismatch distribution), whereas another procedure (BSP) detected population growth from 500,000 to 80,000 ya, with a population decrease around 40,000 ya. (3) Three genetically distinct groups were detected within *G. vittata*. One group was in Central America and northern Colombia, another in French Guiana, and the third in the rest of South America. (4) The origin of Neotropical Lyncodontini was older than in other studies (late Miocene, early Pliocene). (6) Some of the analyses detected a closer relationship between *L. patagonicus* and *G. cuja* than the latter with *G. vittata*. This could show that *Galictis* might be a paraphyletic genus. (7) Many analyses detected that the *L. patagonicus* haplotypes were the first to originate in situ in South America before the definitive closure of the isthmus of Panama, followed by those of *G. cuja*, with *G. vittata* showing the most recent haplotypes. This could indicate that the origin of the current Neotropical Lyncodontini occurred in the south of South America and that colonization occurred towards the north.

## Simple Summary

Carnivorans are of vital importance from a biological conservation perspective because they play a crucial role in the food chains of the ecosystems they inhabit. The Neotropics are home to several families of terrestrial mammalian carnivores (Felidae, Ursidae, Canidae, Procyonidae, Mephitidae, and Mustelidae). Mustelids are the most diverse group, containing the most species of carnivorans worldwide, with the most extreme ecological adaptations. Nevertheless, Neotropical mustelids have not been extensively studied at the population genetic level using genetic markers, and there have been very few molecular studies conducted to reveal their evolutionary history. For this reason, although the number of specimens analyzed for three mitochondrial markers was limited, we present here a molecular phylogenetic study involving the only three species of Neotropical Lyncodontini and two species that have been virtually unstudied at the molecular population genetics level: *Galictis vittata* and *Lyncodon patagonicus*. The results obtained allow us to make some of the first estimates of the level of genetic diversity and genetic heterogeneity among their populations and possible demographic changes throughout the evolutionary history of *G. vittata*. With these results we propose certain changes in the taxonomy of the Neotropical Lyncodontini, as well as possible hypotheses as to how the colonization of South America by this subfamily of mustelids took place.

## 1. Introduction

Carnivorans are vital to the food chains and interconnected networks of most ecosystems [1,2]. Neotropics are recognized for having some of the most important biodiversity hotspots on the planet [3] and therefore contain an abundant carnivoran fauna. Nevertheless, some Neotropical carnivoran species are among the least studied, especially from a genetic perspective. This is the case for Neotropical Lyncodontini [4,5,6,7].

The Mustelidae family, within the order Carnivora, is the largest family within this order, including as many as 65 living species, the species of mustelids being highly diverse in form and size and ecologically very versatile, inhabiting boreal regions and tropical environments [8]. In the last two decades, different phylogenetic studies have been conducted using the molecular study of different types of genes [9,10,11,12] categorizing the 22 mustelid genera into eight primary lineages defining eight subfamilies: Lutrinae (*Aonyx*, *Amblonyx, Enhydra*, *Hydrictis*, *Lontra*, *Lutrogale*, *Lutra*, and *Pteronura*), Mustelinae (*Mustela* and *Neogale*), Ictonychinae (*Galictis*, *Ictonyx*, *Lyncodon*, *Poecilogale*, *Poecilictis,* and *Vormela*), Helictidinae (*Melogale*), Guloninae (*Eira*, *Gulo*, *Martes, Pekania,* and even *Charronia*), Melinae (*Arctonyx* and *Meles*), Mellivorinae (*Mellivora*), and Taxidiinae (*Taxidea*).

Koepfli et al. [8,10] showed that the most basal, and therefore the earliest, branching lineages within the mustelids consist of the three subfamilies of badgers (Taxidiinae, Mellivorinae, Melinae). The next linage to diverge was the subfamily Guloninae, which shows that the tayra (*Eira barbara*; the first split within this subfamily), fisher (*Pekania pennanti*), and wolverine (*Gulo gulo*) occur as successive ancient splits at the base of this clade. Then, the ancestors of the Helictidinae diverged. Subsequently, the ancestors of the subfamily Ictonychinae diverged, whose Neotropical species are of interest in the present work. It is very interesting to emphasize that this subfamily includes species with distributions spread across three continents, Africa (*Poecilictis libyca*, *Ictonyx striatus*, and *Poecilogale albinucha*), Eurasia (*Vormela peregusna*), and South America (*Galictis vittata*, *Galictis cuja*, and *Lyncodon patagonicus*). Interestingly, species within this clade are united by possession of aposematic fur coloration (usually distinctive black and white patterns) and enlarged anal scent glands that are used in defensive threat displays [13]. The ancestors of the subfamilies Mustelinae and Lutrinae were the last to diverge in that order. Alternatively, the phylogenies generated by Fulton and Strobeck [9], Wolsan and Sato [11], Sato et al. [12], Nyakatura and Bininda-Emonds [14], and Hassanin et al. [15] show that after the divergence of the ancestor of Helictidinae, the next ancestor to diverge would be that of Mustelinae and finally Ictonychinae, sister to Lutrinae.

Several phylogenetic studies [8,9,11,12,13,14,15] have also been used to reconstruct the biogeographic history of mustelid lineages and to understand the evolution and assembly of mustelid communities across different continents. One of the most striking findings was that the clades defining the subfamilies Lutrinae, Mustelinae, Ictonychinae, and Guloninae each revealed a deep split that separated a sub-clade composed of New World species (North and/or South America) from a sub-clade composed of Old World species (Africa and/or Eurasia). Perhaps the most relevant of these examples relates to the subfamily (Ictonychinae) of interest in this research. As we discussed earlier, grisons (*Galictis*) and Patagonian Weasels (*Lyncodon*) from South America (Lyncodontini) [12] joined together in a sub-clade that was sister to a sub-clade containing the four species of Old World Ictonychinae.

As we mentioned above, the Neotropical species of the subfamily Ictonychinae stand out as understudied lineages that have undergone remarkable adaptive radiation on the continent [5,16]. Both *Galictis* and *Lyncodon* are considered among the least studied carnivoran genera in the Americas. Even basic aspects, such as species limits of these Ictonychinae and geographical distribution, are still uncertain. Hence, not surprisingly, the acquisition of basic information about *Galictis* and *Lyncodon* has already been cited as a priority for carnivoran studies in the Neotropics [4,17].

Two species, *G. vittata* (Greater Grison) and *G. cuja*, (Lesser Grison), are recognized [18,19,20], although some authors have continued to recognize *Galictis allamandi* (Bell 1841, type locality Suriname) as a potentially valid species [21,22]. Nonetheless, most authors consider *G. allamandi* to be a junior synonym of *G. vittata* [5,19,20].

*G. vittata* occurs at low elevations from southern Mexico throughout Central America into South America as far south as Bolivia, northern Argentina, Santa Catarina state, Brazil, and different points of Paraguay [23,24,25,26,27]. The geographic range of *G. vittata* was estimated at 13,083,600 km^2^ [28]. For example, in the Colombian Atlántico, Bolivar, Casanare, and Santander departments, field observations show that the specimens ranged from 37 to 320 m above sea level (masl) [29]. Nevertheless, the exact geographic distribution of this species is unknown, with numerous contradictions among different authors. This is because few precise specimen records exist [30,31,32,33]. Therefore, the documented distribution has large gaps. Some authors have considered the southern limit of *G. vittata* to be in northeastern Brazil [34,35,36]. On the contrary, others have indicated that it is in southeastern Brazil [37] or southern Brazil [5,19]. Some authors have even denied the existence of this species in Brazil because the same was not included in the list of occurrences for this species in a taxonomic and geographical reference source for mammals [18]. For instance, the presence of the species in Paraguay has been subject to dispute [38]. Traditionally, the Paraná River has long been known to be a dispersal barrier for certain mammal species [39], leading some authors to dismiss the presence of *G. vittata* in Paraguay [19,40], although it has been recorded in a few localities in the Argentinian Misiones province [41,42]. In 2010 [38], a road-killed adult male was found on the Itaipu Superhighway, Alto Paraná department. The natural vegetation of that area is Atlantic Forest, with the immediate area characterized by islands of disturbed forest, pastureland, and isolated human dwellings. The second finding corresponds to the observation of two specimens in 2012 at Km 603 of the Trans-Chaco Highway, Boquerón department. This was in an area of arid Chaco vegetation, heavily modified for cattle ranching. This record extends the known Paraguayan distribution of *G. vittata* approximately 675 km northwestward, well into the Dry Chaco. This species is generally considered typical of forests, but this observation extends its presence to dry areas as well.

Traditionally, four subspecies are recognized [43]: *Galictis vittata andina* (Thomas, 1903:462; type locality Pozuzo, Huánuco department, Peru), *Galictis vittata brasiliensis* (Thunberg, 1820:401; type locality Brazil, South America, restricted by Lonnberg (1921:19) to “probably from Rio de Janeiro” but in any case from southern Brazil; *G. allamandi* and *Galictis crassidens* Nehring, 1885:168; type locality Minas Gerais state, Brazil, are junior synonyms), *Galictis vittata canaster* (Nelson, 1901:129; type locality: Tunkas, northern Yucatán, Mexico), and *Galictis vittata vittata* (Schreber, 1776:447; type locality: Suriname). Although subspecies boundaries are largely unknown, *G. v. andina* could occur in Peru and Bolivia, *G. v. brasiliensis* in southeastern Brazil, *G. v. canaster* in Central America, and *G. v. vittata* in a large fraction of northern South America [43].

*G. cuja* occurs in southern Peru (at high elevations in the altiplano in Arequipa and Puno departments) [44], Bolivia (Andean highlands, east slopes of the Andes, and southeastern Bolivia) [19,23,45,46,47], Chile (from Arica southward and from sea level to 3800 m in central Chile, from Coquimbo to Valdivia provinces) [20,48,49], Paraguay (in the xeric Chaco and probably throughout the country) [38,50,51], Uruguay (throughout all of the country) [52], Argentina (a major fraction of the country, including more than 50° S) [53,54], and east to southeastern Brazil [5,6,20]. As with *G. vittata*, confusion surrounds the distribution of *G. cuja*, with some sources listing its occurrence as far north as northeastern Brazil [20], while other authors indicate its northernmost limit on the Atlantic coast in southeastern Brazil [37]. This species often inhabits drier and higher environments than *G. vittata*, inhabiting a great variety of habitats from sea level to 4200 masl, such as arid scrub, the Chaco Desert, steppes, wet forest, and Andean shrublands [20]. In Brazil, this species occupies a wide variety of diverse environments, including the Cerrado and Caatinga in the northeast, as well as the tropical and subtropical moist broadleaf forest (Atlantic Forest) throughout the eastern seaboard and the Uruguayan savanna towards southern Brazil [5].

Some authors place *G. cuja* in a separate genus (or subgenus), *Grisonella* [48,55]. Traditionally, four subspecies are recognized [43]: *Galictis cuja cuja* (Molina, 1782:291, type locality Chili; Thomas 1912:46, restricted to southern Chile, Temuco, and later restricted by Cabrera 1958: 261 to the Santiago area, where Molina made his principal observations), *Galictis cuja furax* (Thomas, 1907:162; type locality San Francisco dos Campos, southern Minas Geraes state, altitude 1580 masl, Brazil), *Galicitis cuja huronax* (Thomas, 1921a: 213, type locality Mar del Plata, Buenos Aires province, Argentina), and *Galictis cuja luteola* (Thomas, 1907:163; type locality Chulumani, Bolivia, 67° W, 16° S, altitude 1800 masl).

The Patagonian Weasel (*L. patagonicus*) lives in the Southern Cone of South America (Argentina and Chile), being one of the most poorly known carnivorans of South America [22,56,57]. This small mustelid is known from a few direct observations and collected specimens. *Lyncodon* is almost entirely restricted to Argentina, and indications from Chile are limited to two authors [58,59]. Most historical records were made in the nineteenth and early twentieth centuries. For example, the last confirmed record from Buenos Aires province was made in 1926 [60]. Today, this species has probably disappeared from almost the entire province of Buenos Aires and potentially could survive south to Bahía Blanca and in the boundary with the La Pampa province. An increase in precipitation of more than 300 mm per year in recent decades and the expansion of agriculture are considered the main factors triggering the retraction of the Patagonian Weasel in Buenos Aires province [56]. The locations where this species has been observed in the last 20 years have been in Santa Cruz, Chubut, Río Negro, and San Juan provinces in Argentina [57]. For example, the *L. patagonicus* specimen used by Sato et al. [12] and Wolsan and Sato [61,62] came from Puerto Madryn (Chubut province) in Argentine Patagonia.

Cabrera [63], with extensive knowledge of the Neotropical mammal fauna, distinguished two subspecies within *L. patagonicus* [41,59]. These subspecies are *Lyncodon patagonicus patagonicus* (de Blainville, 1842), in Patagonia, southern Buenos Aires, and Mendoza provinces, and *Lyncodon patagonicus thomasi* (Cabrera, 1929), from northwestern Argentina. For the moment, this has not been accepted by most authors until further research can be carried out on the matter.

Thomas [64,65] was the first author to attempt to delineate morphological characteristics to determine how many different taxa of grisons existed in the Neotropics. In fact, Thomas was the first to recognize the diagnostic characteristics that are still perceived as valid today to differentiate *G. vittata* and *G. cuja* [19,20]. He noted that specimens from the Brazilian Minas Gerais state lacked an internal cusp in their first lower molar that seemed to be present in grisons from northern South America. Subsequently, he proposed that there were two forms of grisons: a larger one presenting this internal cusp in the first lower molar (and occurring mostly in the northern Neotropics), and a smaller one lacking this internal cusp and inhabiting more southerly areas of South America [64]. Furthermore, *G. vittata* is consistently larger than *G. cuja*. Similarly, there are other differentiating characteristics between the two *Galictis* species. *G. vittata* shows a proportionally shorter tail (around 30%) than *G. cuja* (around 40%) and fewer tail vertebrae (17–18 vertebrae vs. 20–21 vertebrae). The tips of dorsal guard hairs are generally white to gray in *G. vittata* and buffy to yellowish in *G. cuja* [19,21]. Calcanea of *G. vittata* and *G. cuja* differ mainly in size [66]. *Galictis vittata* is significantly larger than *G. cuja* in body size for many physical characteristics (total length: 600–760 mm vs. 443–680 mm; length of hind foot: 66–97 mm vs. 50–75 mm; body weight: 1.5–3.8 kg vs. 1.2–2.5 kg; width across canines: 18–24 mm vs. 13–19 mm; condyle–basilar length: 80–98 mm vs. 64–84 mm). Bacula of *G. vittata* and *G. cuja* are similar in shape, but the shaft is longer (54–57 mm vs. 41–47 mm) and wider (2–3 mm vs. 1–2 mm), the tip is proportionately longer (6–8 mm vs. 5–6 mm), and knobs are more prominent in *G. vittata* [67,68]. *G. cuja* shows a denser and/or longer pelage, leading to a “furrier” appearance; meanwhile, this feature is never observed in *G. vittata*, whose fur is always shorter [5]. *Lyncodon patagonicus* is like *G. vittata* but considerably smaller, but the top of its head is white or creamy; there are long white hairs on the dorsum and throat, and the sides are dark brown rather than black [19,21]. Its feet are webbed only a short distance beyond the plantar pads, and P2 and p2 are missing from its dentition [69,70]. In terms of size, *L. patagonicus* is more like *G. cuja* (35.5–43 cm and 1.5 kg vs. 27–52 cm and 1.3–2.4 kg), but certain differences with respect to *G. cuja* are very similar to those described with respect to *G. vittata* (top of head is white or creamy, throat and sides are dark brown rather than black, long white hairs are present on the dorsum, etc.). Additionally, the tympanic bulla is more inflated in *L. patagonicus* [70,71,72,73].

Most molecular studies that have been carried out with *Galictis* and *Lyncodon* species have involved phylogenetic but not population genetic issues. Koepfli and Wayne [74] evaluated the phylogenetic utility of five nuclear gene segments (amplified with Type I sequence-tagged site primers or STS) against the mt*Cyt-b* gene. The main objective was to resolve evolutionary relationships within the Mustelidae family, showing that nuclear markers showed fewer homoplasies than mt*Cyt-b*, including one specimen of *G. vittata* from Peru. Fulton and Strobeck [9] analyzed 79 carnivoran species, with 20 genera and 36 species of mustelids (including *Galictis vittata*) and five nuclear markers totaling 2974 base pairs (bp). Koepfli et al. [10] analyzed the molecular phylogeny for most mustelid genera (21 of 22) (and a good number of species, 43) using segments from 22 different genes (21 nuclear genes and the complete mt*Cyt-b* gene; total sequenced approximately 12,000 base pairs, bp). In that study, one specimen of *G. vittata* (the one previously used in [74]) and one specimen of *G. cuja* (from Argentina) were sequenced for these genes, and no *L. patagonicus* samples were included in the analysis due to the difficulty of obtaining samples of this species. Harding and Smith [75] analyzed mt*Cyt-b* gene sequences to reassess the phylogeny of American mustelids. The study concluded that the American mink and the weasels endemic to the Americas form an independent phylogenetic lineage shaped by historical biogeographical events. They used the sequences from the two previous studies for one specimen of *G. vittata* and one from *G. cuja*. Agnarsson et al. [76] presented a phylogeny based on the mt*Cyt-b* gene of 222 extant and 4 extinct carnivore species. The study confirmed the division into Caniformia and Feliformia but noted significant conflicts between traditional morphological classification and molecular evolutionary relationships (including one specimen of *G. vittata* and one specimen of *G. cuja*). Wolsan and Sato [11] analyzed 52 musteloid species, with 22 genera and 43 species of mustelids (including *Galictis vittata*) and 25 nuclear and 29 mitochondrial markers, totaling 27,965 bp. Nyakatura and Bininda-Emonds [14] reconstructed a comprehensive species-level supertree encompassing all living carnivores on the planet. The analysis included sequences from one specimen of *G. vitatta* and another of *G. cuja*. By compiling both morphological characteristics and nuclear and mitochondrial molecular sequences, the authors combined the evolutionary data of South American mustelids with those of 284 other carnivore species. This allowed them to pinpoint their divergence times and their exact position within the subfamily Guloninae. Sato et al. [12] also attempted to resolve the phylogeny of mustelids (39 species and 18 genera) by analyzing nine nuclear genes and the mt*Cyt-b* gene (total 8400 bp). The study included one specimen of *G. vittata* and one of *G. cuja*, and, for the first time, analyzed a specimen of *L. patagonicus*. Kocher et al. [77] used five specimens of the *G. vittata* from French Guiana to evaluate the effectiveness of mt*12S rRNA* metabarcoding in the identification of Amazonian mammals. Bornholdt et al. [5] performed an extensive morphological analysis and a more moderate molecular analysis of the genus *Galictis*. That was the first population genetic study with a species of Neotropical grison, specifically *G. cuja*. The authors analyzed ten specimens of *G. cuja* (five from Brazil and five from Argentina) and three specimens of *G. vittata* (from Peru but without specific geographic locations) using the mt*NADH5* gene and were able to significantly differentiate between the two species. Bontempo et al. [6] analyzed 28 specimens of *G. cuja* in east–central Brazil for the mt*Cyt-b* gene for the purpose of population genetic analysis. Hassanin et al. [15] incorporated a complete mitogenome of *G. vittata* to reconstruct the evolutionary history of carnivores. This analysis placed *Galictis* as one of the most basal lineages within the Ictonychinae subfamily. Nonetheless, the mitogenomes of *G. cuja* or *L. patagonicus* were not included. Sato and Wolsan [78] showed at higher taxonomic levels (among genera or older) within the order Carnivora that nuclear DNA was less homoplastic than mitochondrial DNA and therefore better suited for studying deep-level relationships using supermatrix or supertree methods. The study showed that the red panda, *Ailurus fulgens*, studied using nuclear DNA, Bayesian and maximum likelihood phylogenetic inference, and the supermatrix approach contributed new knowledge to the phylogenetic position of this species. The study also showed that it clarified the evolution of Mustelidae, especially from the Mustelidae subfamilies. Wolsan and Sato [61] used sequences from a specimen of *G. cuja* and a specimen of *L. patagonicus*, revealing that the Lyncodontini tribe functionally lost the umami taste receptor (*TAS1R1*–*TAS1R3*) due to past dietary specialization. Despite being obligate carnivores today, their semi-aquatic ancestors disabled this gene, demonstrating how extreme dietary transitions leave permanent genetic scars. Filippini [79] analyzed 21 specimens of *G. cuja* in Argentina for the mt*NADH5* gene for the purpose of population genetic analysis. Finally, Wolsan and Sato [62] analyzed the full-length coding sequences of the *TAS1R1* and *TAS1R3* genes of *L. patagonicus* and *G. cuja* in search of inactivating mutations that convert functional genes into nonfunctional pseudogenes. The DNA sequence data for *TAS1R1* and *TAS1R3* of *L. patagonicus* and *TAS1R1* of *G. cuja* were used. There were two nonsense and two frameshift mutations in *TAS1R1* of *L. patagonicus*, one nonsense and two frame shift mutations in *TAS1R1* of *G. cuja*, and one nonsense mutation in *TAS1R3* of *L. patagonicus*. While the nonsense mutations were species-specific, the frameshift mutations were identical between both species. The *TAS1R1*–*TAS1R3* umami (savory) taste receptor lost its function in the Lyncodontini’s stem lineage around 3 to 9.5 million years ago, mya. This finding was intriguing because Lyncodontini apparently need this receptor (they are terrestrial carnivores with diets high in umami-eliciting compounds, including purine 5′-monophosphate ribonucleotides, the main agonists of *TAS1R1*–*TAS1R3* genes in carnivorans). The authors hypothesized that a common ancestor of extant Lyncodontini lost *TAS1R1*–*TAS1R3* function and this ancestor was semi-aquatic and predated mainly on fish and/or aquatic invertebrates (tissues of living or recently dead fish and aquatic invertebrates are low in purine 5′-monophosphate ribonucleotides), which is consistent with the idea that loss of taste receptor function is caused by feeding specializations and that a prolonged semi-aquatic episode in the evolutionary history of the Lyncodontini’s stem lineage was present.

Regarding the generation of mitochondrial sequences, several interesting sequences of two mt genes (*NADH5* and *Cyt-b*) have been generated for *G. cuja*. Nonetheless, at the mtDNA level, only two mt*Cyt-*b gene sequences and three mt*NADH5* gene sequences have been generated for *G. vittata*, while only one mt*Cyt-b* gene sequence has been generated for *L. patagonicus*. Therefore, very few population genetic studies have been conducted only for *G. cuja*, but no such studies have yet been published on *G. vittata* and *L. patagonicus*.

Taking everything said into consideration, mtDNA analysis in recent decades has made it possible to overcome the limitations of morphological phylogenies by providing informative markers to resolve phylogenetic relationships, estimate population genetic parameters, and reconstruct the evolutionary history of a given biological group [80,81,82]. Mitochondrial genes are powerful markers for phylogenetic tasks because they lack introns and include a rapid accumulation of mutations, rapid coalescence time, no recombination rate, and haploid inheritance [80]. For all of these reasons, mt gene trees are more precise in reconstructing the history of divergence among related taxa than other molecular markers [81]. Supporting this, Cummings et al. [82] showed that mt genomes have greater information content per base than nuclear DNA. Nonetheless, the mt genes pose several problems, including heterogeneity in base composition at each codon position and third-codon position saturation. Jointly, care should be taken when using mt genes for resolving taxonomic problems because gene trees do not necessarily correspond well with species trees [83]. Moreover, mt data show only the evolution of the female lineages, and this could miss hybridization events between close species when males are the gene flow vectors (“mitochondrial capture”) [84].

Nevertheless, mt genes present an advantage, as they amplify more easily than nuclear genes, given that cells carry a high number of copies of mtDNA molecules, in the face of a serious drawback encountered at the beginning of this research. The samples obtained from the three Neotropical Lyncodontini species, collected over the last 25 years for the present study, consisted mainly of a few hairs or very small fragments of skin or bone, and the DNA obtained was scarce and of low quality and therefore unsuitable for obtaining mitogenomes or nuclear gene sequences. Because of this, we were forced to amplify very small fragments of some mt genes. But which mt genes would be suitable? And could these small fragments of mt genes offer phylogenetic results like those obtained in exhaustive studies previously conducted to resolve the phylogeny of mustelids? [8,9,10,11,12,13,14,15,61,62].

Based on the above, we selected three small fragments of mtDNA. One corresponds to the mt*NADH5* gene (a gene with a medium-high evolution rate), one to the mt*12S rRNA* gene (low evolution rate), and one to the control region (*D-loop*) (very high evolution rate). These three fragments could thus represent the average evolution rate of mtDNA. We must remember that although mtDNA represents a single linked locus, selection pressures and evolutionary rates are highly heterogeneous across it [85,86]. Thus, we report 13 new mt*NADH5* gene sequences, 11 new mt*12S rRNA* gene sequences, and four new mt*D-loop* sequences for *G. vittata* and new sequences (from one or two specimens) of *L. patagonicus* for those same three markers.

As a consequence, the main aims of the research presented here are as follows: 1—to estimate the levels of mitochondrial genetic diversity of *G. vittata*; 2—to estimate whether there is any type of specific genetic structure within *G. vittata*; 3—to determine possible demographic changes throughout the evolutionary history of *G. vittata*; 4—to show whether the small fragments of mtDNA chosen yield phylogenies of different genera of Neotropical mustelids similar to those found with multigenetic data collections generated by other authors [8,9,10,11,12,13,14,15,61,62]; 5—to estimate the divergence times between the three species of Neotropical Lyncodontini species and other genera of Neotropical mustelids; and 6—to reconstruct the evolutionary history of living species of Neotropical Lyncodontini and other genera of Neotropical mustelids from the Miocene to the present.

## 2. Materials and Methods

### 2.1. Samples

The following samples were obtained for DNA extraction. For *G. vittata*, 13 samples were used from Guatemala (1), Colombia (2), Ecuador (2), Peru (4), Bolivia (1), and French Guiana (3). For *G. cuja*, two samples were used (Argentina). For the mt*NADH5* gene analysis, six additional sequences from *G. cuja* for this gene obtained from GenBank from the study by Bornholdt et al. [5] were used because those sequences had precise geographical origins. For *L. patagonicus*, two samples were used (Argentina). Additionally, in the phylogenetic analyses and divergence time estimation, two specimens of Tayra (*Eira barbara*) from French Guiana, two specimens of Long-Tailed Weasel (*Neogale frenata*) (one from Colombia and one from Ecuador), two specimens of Neotropical Otter (*Lontra longicaudis*) from Peru, one specimen of Southern River Otter (*Lontra provocax*) from Chile, and one specimen of Marine Otter (*Lontra felina*) from Chile were analyzed. The exact geographic origins and the type of tissue used for DNA extraction are shown in Table 1.

### 2.2. Mitochondrial DNA

The samples included a few hairs per sample and small fragments of skin and bone. Each tissue type was washed with SDS and distilled water, followed by a rinse with absolute ethanol and drying at 50 °C to remove as many contaminants and Dnases as possible before DNA extraction. For preparation, skin samples were cut into very small fragments, while hair samples were treated with 10% Chelex. Bone samples were pulverized using a Dremel tool. DNA from skin and bones were obtained using the NX-48S Tissue 261 DNA kit (NextractorR NX-48S; Genolution, Seoul, Republic of Korea).

PCR amplification was performed in 25 μL reactions containing 10–100 ng/μL of genomic DNA using validated primers for the mt*NADH5* (L12673: 5′ GGTGCAACTCCAAATAAAAGTA-3′, H12977: 5′-AGAATTCTATGATGGATCATGT-3′) [73] (265 bp), mt*12S rRNA* (L1091: AAAAAGCTTCAAACTGGGATTAGATACCCCACTAT-3′; H1478: 5′- TGACTGCAGAGGGTGACGGGCGGTGTGT-3′) [87,88,89] (345 bp), and mt*D-loop* (MTLPRO2 forward primer: 5′-CACTATCAGCACCCAAAGCTG-3′, designed by Tchaicka et al. [90], and the reverse primer LonCR-R1: 5′-ATGGTTTCTCGAGGCATGGT-3′, designed by Trinca et al. [91], 226 bp) markers in combination with a standard mix and OneTaq 2x Master Mix (New England Biolabs, Ipswich, MA, USA), following a standard thermocycling protocol beginning with initial denaturation. The PCR temperatures were 95° for 5 min, followed by 40 cycles of 1 min at 94 °C, 1 min at 52–56 °C (depending on the primers used) and 1 min at 72 °C, and one final extension of 10 min at 72 °C. All amplifications, including positive and negative controls, were checked in 2% agarose gels and stained with Safe Green. Those samples amplified were purified using membrane-binding spin columns (QIAquick PCR Purification Kit; Hilden, Germany). The PCR products were sequenced in both directions using the Big Dye™ kit in an ABI 377A automated DNA sequencer (Thermo Fisher Scientific, Waltham, MA, USA). A consensus of the forward and reverse sequences was determined using Sequencher software v.5.4.6 (Gene Codes Corporation, Ann Arbor, MI, USA).

### 2.3. Statistical Analyses

#### 2.3.1. Genetic Diversity and Genetic Heterogeneity Statistics and Possible Demographic Changes in *Galictis vittata* for Three Mitochondrial Markers

Each of the analyses that were carried out were performed separately for each of the three mt molecular markers used (*NADH5*, *12S rRNA*, *D-loop*), as the sample size is different for each of the genetic markers used and to observe whether each of the genes that possesses different rates of evolution nevertheless offers compatible results with each other. The following genetic diversity statistics were applied to the three mt markers to determine the genetic diversity of *G. vittata*: number of haplotypes (H), haplotype diversity (H_d_), nucleotide diversity (π), and θ statistic by sequence. These genetic diversity statistics were calculated with DNAsp v6.0 and Arlequin v3.5.1.2 software [92,93].

For the mt*NADH5* and the mt*12S rRNA* genes, we estimated the following statistical heterogeneity indices for the overall *G. vittata* sample: table of contingency, H_ST_, K_ST_, K_ST*_, γ_ST_, N_ST_, and F_ST_ [94]. Indirect gene flow estimates were obtained assuming an infinite island model [95]. Significance was estimated with permutation tests using 10,000 replicates. We also estimated genetic heterogeneity between *G. vittata* population pairs. For this task, we used exact probability tests with Markov chains using 10,000 dememorization parameters, 20 batches, and 5000 iterations per batch. All of the heterogeneity statistics were calculated with DNAsp v6.0 and Arlequin v3.5.1.2 software [92,93].

To determine the degree of genetic differentiation between the different mustelid species analyzed, the Kimura 2P genetic distance was used [96], which quantifies the percentage of genetic differences within the different groupings detected within *G. vittata* and between the different mustelid species analyzed.

For the mt*NADH5* and the mt*12S rRNA* genes, we conducted a Bayesian Analysis of Population Structure (BAPS) with the software BAPS v6.0 [97] using mixed and unmixed models to group genetically similar individuals into panmictic genetic clusters. Calculations were performed, with the number of k clusters varying from 2 to 20. Five replicates were carried out for each k value. A BAPS analysis was also performed on the same two genes to see if it was possible to perfectly distinguish the three species of Neotropical Lyncodontini studied using the same conditions just described.

To estimate possible demographic changes for *G. vittata* at the mt*NADH5,* mt*12S rRNA*, and mt*Dloop* genes, we used Fu and Li’s D* and F* tests [98], Fu’s F_S_ statistic [99], Tajima’s D test [100], and R_2_ statistic [101]. Ninety-five percent confidence intervals and probabilities were obtained with 10,000 coalescence permutations. Also, a mismatch distribution (pairwise sequence differences) was obtained [102]. We used raggedness (*rg*) to determine the similarity between the observed and theoretical curves. These demographic analyses were also carried out using DNAsp v6.0 and Arlequin v3.5.1.2 software [92,93]. Using the mismatch distribution test, the parameter τ (τ = 2μt) was obtained, where μ is the mutation rate per generation and *t* is the number of generations since population expansion began. If a generation in *G. vittata* is about 6 months (although it is not strictly seasonal), it was estimated how long ago a demographic change could have occurred in this species.

An additional procedure was used to estimate possible population changes throughout the natural history of *G. vittata* using the mt*NADH5* and mt*12S rRNA* genes. For this, we used the coalescence-based Bayesian skyline plot (BSP). BSP analysis was performed in BEAST v2.4.3 using the empirical base frequencies and a strict molecular clock [103,104]. We applied jModelTest v2 to evaluate the best substitution models. Additionally, we assumed a substitution rate of 7 × 10^−8^ substitutions per site and per year to obtain the time estimates in years as well as with kappa with log-normal (1, 1.25) and a skyline population size that was uniform (0, infinite; initial value, 80). We conducted a total of five independent runs of 40 million Markov chain Monte Carlo (MCMC) iterations following a burn-in of 10% of iterations, logging every 10,000 iterations. We selected a stepwise (constant) Bayesian skyline variant with maximum time as the upper 95% high posterior density (HPD). The total height of the tree was determined by treeModel.rootHeight. The convergence of the analysis was assessed by checking the consistency of the results over five independent runs. For each run, we used the software Tracer v1.7 to inspect the trace plots for each parameter to assess the mixing properties of the MCMCs and to estimate the effective sample size (ESS) value for each parameter. Runs were considered as having converged if they displayed good mixing properties and if the ESS values for all parameters were greater than 200. We discarded the first 10% of the MCMC steps as a burn-in and obtained the marginal posterior parameter distributions from the remaining steps using Tracer v1.7. To test whether the inferred changes of effective numbers over time were significantly different from a constant population size null hypothesis, we compared the BSP obtained with the “Coalescent Constant Population” model (CONST) implemented in BEAST v2.4.3 using Bayes Factors. Therefore, we conducted five independent CONST runs using 40 million MCMC iterations after a burn-in of 10%, logging every 10,000 iterations. We assessed the proper mixing of the MCMC and ensured that ESSs were greater than 200. We then used the Path sampler package in BEAST v2.4.3 to compute the log of marginal likelihood (logML) of each run for both BSP and CONST. We set the number of steps to 100 and used 40 million MCMC iterations after a burn-in of 10%. Bayes Factors were computed as twice the difference between the log of the marginal likelihoods (2[Log ML(BSP) – Log ML(CONST)]) and were performed for pairwise comparisons between BSP and CONST runs. As recommended, Bayes Factors greater than 6 were considered strong evidence to reject the null hypothesis of constant effective numbers throughout time. Nevertheless, all of these demographic procedures have several caveats. Selection can affect the effective population size, reducing the effective number for a time and increasing the coalescence rate later [105]. The same occurs with small changes in the mutation rate (µ), which can greatly affect the effective number and, in turn, estimated divergence time [106].

#### 2.3.2. Phylogenetic Trees, Haplotype Networks, and Divergence Times

jModeltest v2.0 [107], Kakusan4 [108], and MEGA X v10.0.5 [109] software were applied to determine the best evolutionary mutation model for the analyzed sequences of the three markers analyzed. The Bayesian information criterion (BIC), the Akaike information criterion (AIC), and the maximum likelihood criterion (lnL) [110,111,112] were used to determine the best evolutionary model for the three mt markers used for the analysis of the different genera of mustelid studied. We generated maximum likelihood (ML), Bayesian inference (BI) and DensiTree (DT) trees for each one of the three mt markers analyzed. For the ML tree, we used RAxML v8.2.12 software [113]. RAxML was run with the GTRGAMMA model of nucleotide evolution and default parameters and 1000 bootstrap replicates to calculate node support. BI trees were completed with BEAST v. 2.4.3 software [103]. Four independent iterations were run with 10 MCMC chains sampled every 10,000 generations for 20 million generations after a burn-in period of 4 million generations. We checked for convergence using Tracer v1.7.2 [114]. We plotted likelihood versus generation and estimated ESS > 200 of all parameters across the four independent analyses to determine convergence and optimal results. The results from different runs were combined using LogCombiner v1.10.4 software [115] and TreeAnnotator v1.10.4 [116]. A Yule speciation model and a relaxed molecular clock with an uncorrelated log-normal rate of distribution were used [117]. Posterior probability (*pp*) values provide an assessment of the degree of support of each node on the tree. Trees were visualized in FigTree v. 1.4.4 software [118]. These BI trees, obtained with BEAST v. 2.4.3 software, were used to estimate the time to most recent common ancestor (TMRCA) for the different nodes found with BI trees. We used 11.8 million years (mya) as a baseline at the root of the tree. This was the value found by Koepfli et al. [10] in their exhaustive study of 22 genes (21 nuclear and 1 mitochondrial) for the origin of the ancestor that gave rise to the current *E. barbara*, which is the oldest Neotropical mustelid genus branch we used in this study.

The DensiTree procedure (DensiTree v. 3.0.2 software [119]) was used. This procedure draws all trees of a determined analysis, but instead of using opaque lines, it uses transparency. In places where many trees have similar topologies and branch lengths, there will be many lines drawn, and the tree figure yields a densely colored area. Places that have a few competing topologies will be highlighted by a web of lines. Uncertainty in node heights and their distribution can be shown by smears around the mean node height. Therefore, the DensiTree procedure provides a qualitative approach to analyzing phylogenetic trees.

The previous BI temporal estimates we obtained belong to one of two different approaches for inferring divergence times [120]. The first approach is based on fossil-calibrated DNA phylogenies or, failing that, using molecular estimates previously established with reliability by other genetic studies. This is the case for our BI trees, where the divergence estimates between the ancestor of *E. barbara* (the most ancient branch) and the ancestors of the other mustelid taxa analyzed (which come from more recent branches) were used. The root age estimate for the BI trees was 11.8 mya, as we mentioned earlier. The second approach is named “borrowed molecular clocks” and uses direct nucleotide substitution rates inferred from other taxa. For this second approach, we used a Median Joining Network (MJ network) with the help of Network 4.6.10 software from Fluxus Technology Ltd. (Essex, UK) [121]. The ρ statistic [122] was estimated and transformed into years of divergence among the haplotypes. To determine the temporal splits, we estimated the mutation rate per sequence and per million years. A nucleotide substitution rate of 1.22% per million years was applied to mt*NADH5* (equivalent to one mutation every 309,310 years), another of 0.42% per million years to mt*12S rRNA* (equivalent to one mutation every 688,137 years), and another of 4% per million years to mt*D-loop* (equivalent to one mutation every 110,619 years). In the case of the first two markers, the evolution rates per million years are well-estimated in carnivorans and the range of oscillation is small (for mt*NADH5* between 1.15% and 1.22% per million years [123] and for mt*12S rRNA* between 0.42% and 0.57% per million years [124]). In the case of the evolution rate per million years for the mt*D-loop* marker, the situation is more complex, as values for different taxa have been reported ranging from 1.5% per million years to 17.75% per million years for certain fox species [125]. After several simulations, the evolution rate per million years that best fit the observed values for the other two markers was around 4% per million years. Networks are more appropriate for intraspecific phylogenies than tree algorithms because they explicitly allow for the co-existence of ancestral and descendant haplotypes, whereas trees treat all sequences as terminal taxa [126].

## 3. Results

### 3.1. Mitochondrial Genetic Diversity, Genetic Heterogeneity, and Possible Demographic Changes in Galictis vittata

Analysis of mitochondrial genetic diversity in *G. vittata* revealed differences in the number of haplotypes and variability estimates among the three mt markers evaluated (Table 2) correlated with the rate of evolution of each of these markers. The mt*NADH5* marker showed the highest number of haplotypes (H = 8), along with the highest value of haplotype diversity (H_d_ = 0.88 ± 0.07) and the second highest value of nucleotide diversity (π = 1.46 ± 0.29; all of the values of π in %). The *mt12S rRNA* locus registered three haplotypes (H = 3), with low haplotype diversity (H_d_ = 0.35 ± 0.17) and reduced nucleotide diversity (π = 0.15 ± 0.08). Similarly, mt*D-Loop* exhibited three haplotypes (H = 3), with high haplotype diversity (H_d_ = 0.83 ± 0.22) and the highest nucleotide diversity value among the analyzed markers (π = 2.08 ± 0.5). The estimates of H and H_d_ may be more affected by the sample sizes used, but the π estimates are unbiased with respect to these sample sizes. Therefore, there was a perfect fit between the π values and the rate of evolution per million years for each of these three mt markers (*D-loop* is the fastest evolving and has the highest π value, while *12S rRNA* has the slowest evolutionary rate and the lowest π value).

Using the total sample of *G. vittata* analyzed for the mt*NADH5* gene, in which the presence of three different populations was detected (see below), it was observed that all of the statistics used to globally detect genetic heterogeneity were highly significant (Table 3). For example, the statistics γ_ST_ = 0.67 (*p* = 0.0004) (gene flow, Nm = 0.24) and F_ST_ = 0.66 (*p* = 0.0005) (Nm = 0.25) showed a very high level of internal genetic differentiation in *G. vittata*. All genetic heterogeneity statistics were also significant among the three groupings detected within *G. vittata* by population pairs and with the genetic distances used (Table 4). The population pair that showed the most differentiated genetic heterogeneity values with γ_ST_ and with the Kimura 2P genetic distance was between the French Guiana grouping and the Central American + northern Colombia grouping, while for F_ST_ the two most differentiated groupings were the French Guiana grouping and the rest of South America. For the mt*12S rRNA* gene, significant genetic heterogeneity was also detected (Table 5) for the two groups within *G. vittata* we used in this analysis (central America + northern Colombia vs. the rest of South America), as we will discuss below. The genetic heterogeneity statistics γ_ST_ = 0.80 (*p* = 0.001) (Nm = 0.12) and F_ST_ = 0.67 (*p* = 0.001) (Nm = 0.25) were significant. The genetic differentiation magnitudes were very similar to those found for the mt*NADH5* gene. Nevertheless, the Kimura 2P genetic distance was smaller than that found for the former gene (Da = 0.29 vs. 1.3–2.4 in %). This latter difference is consistent with the fact that the evolutionary rate of the mt*NADH5* gene is higher than the evolutionary rate of the mt*12S rRNA* gene because a genetic distance is an absolute value while γ_ST_ or F_ST_ are relative statistics.

**Figure 1:**
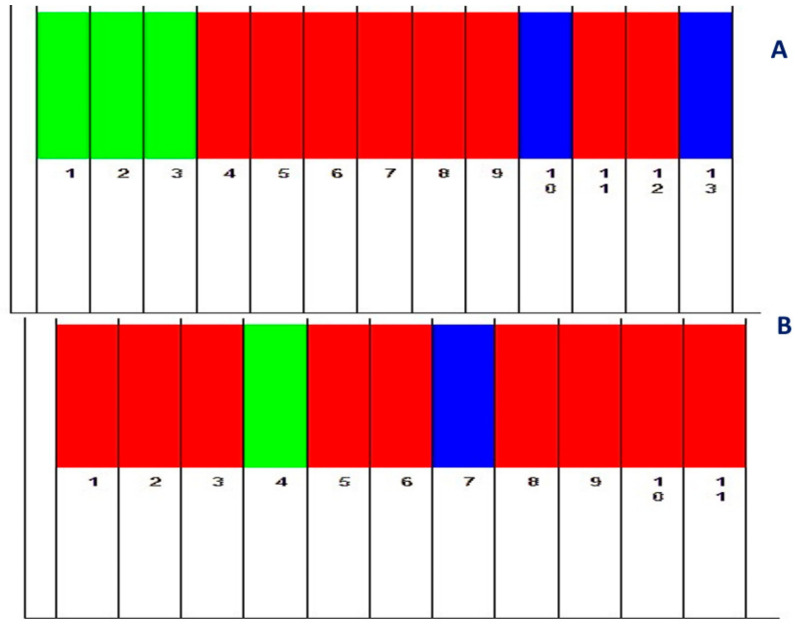
Different populations or taxa of *Galictis vittata* were detected by means of BAPS. Each different color indicates a different population or taxa. (**A**) A sample of 13 specimens of *Galictis vittata* sequenced at the mt*NADH5* gene. Blue: 10 = Jalapa, Guatemala; 13 = Colosó, Sucre, Colombia; green: 1–3 = Roura, Régina, and Camopi in French Guiana; red: 4–9 and 11,12 = rest of South America (Meta, Colombia; Pastaza and Morona Santiago, Ecuador; Loreto and Ucayali, Peru; Cochabamba, Bolivia). (**B**) A sample of 11 specimens of *Galictis vittata* sequenced at the mt*12S rRNA* gene. Blue: 7 = Colosó, Sucre, Colombia; green: 4 = Jalapa, Guatemala; red: 1–3 = Roura, Régina, and Camopi in French Guiana; 5–6 and 8–11 = Meta, Colombia; Pastaza and Morona Santiago, Ecuador; Loreto and Ucayali, Peru; Cochabamba, Bolivia.

The BAPS analysis with the mt*NADH5* gene (Figure 1A) detected three different populations within *G. vittata* [Log (marginal likelihood) of optimal partition = −76.62, *p* = 0.98]. The first grouping comprised specimens from part of Colombia, Ecuador, Peru, and Bolivia. The second grouping included the three specimens from French Guiana; meanwhile, there were two specimens in the third grouping, one from Guatemala and the other from Colosó, Sucre department, in northern Colombia. The BAPS analysis with the mt*12S rRNA* gene (Figure 1B) also detected three different populations within *G. vittata* [Log (marginal likelihood) of optimal partition = −6.14, *p* = 0.72]. In this case, the first grouping contained all of the specimens from South America (part of Colombia, Ecuador, Peru, Bolivia, and French Guiana; this gene did not differentiate the samples from this latter country from those from the rest of South America as the mt*NADH5* gene had done); the second grouping consisted of the specimen from Guatemala, and the third grouping included the specimen from Colosó (Sucre) in northern Colombia.

**Figure 2:**
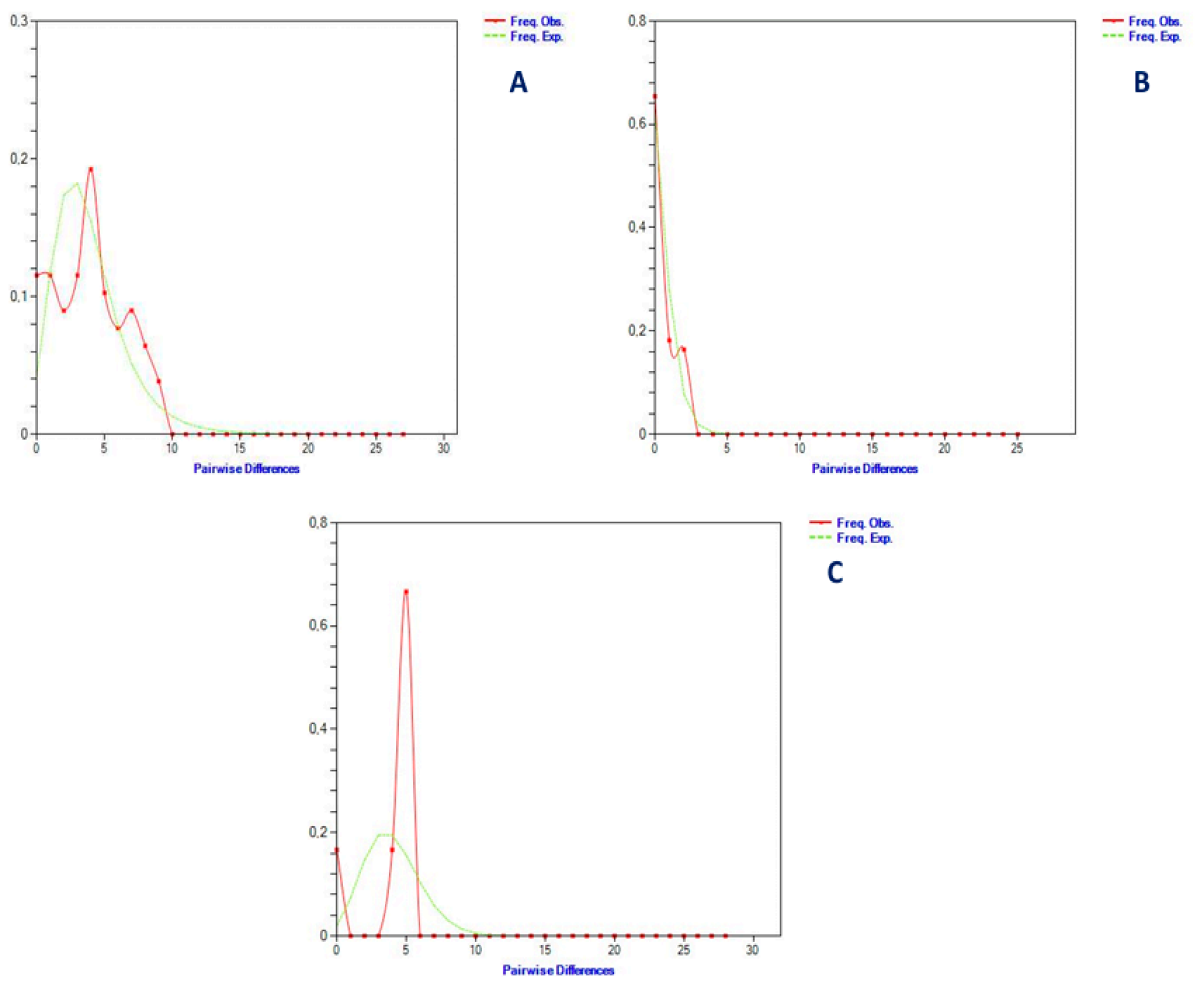
Analysis of mismatch distributions (pairwise sequence differences) at three mitochondrial markers for *Galictis vittata*. (**A**) At the mitochondrial *NADH5* gene; (**B**) at the mitochondrial *12S rRNA* gene; (**C**) at the mt*Dloop* marker. The first analysis was related to a significant female population expansion.

For the mt*NADH5* gene, the mismatch distribution test and the R_2_ statistic were significant, indicating a possible population expansion for *G. vittata* within the analyzed geographic range (Table 6 and Figure 2A). For the other two genes studied, none of the applied tests were significant, although, for example, the visual fit between the observed and expected frequencies for the mismatch distribution test was very similar (Figure 2B,C). The mt*NADH5* gene was the one for which the largest number of sequences was studied, which facilitated the detection of possible demographic changes in the studied species, while in the other two cases the smaller sample sizes prevented obtaining significant values. Furthermore, the mismatch distribution plot for the mt*NADH5* gene showed three peaks corresponding to the three distinct populations found with the BAPS analysis, while the mismatch distribution plot for the mt*12S rRNA* gene suggests two distinct populations within *G. vittata*.

Probably, if the sample sizes for the other two markers had been larger, more evidence of population expansion in *G. vittata* could have been obtained. The t values obtained were 1.436 for mt*NADH5*, 1.107 for mt*12S rRNA*, and 4 for mt*Dloop*, respectively. Using the evolution rates discussed for NJ networks (1.22% for mt*NADH5*, 0.42% for mt*12S rRNA*, and 4% for mt*Dloop*), the onset times of population expansion could have been approximately 370,000 years ago (mt*NADH5*), 635,000 years ago (mt*12S rRNA*), and 369,000 years ago (mt*Dloop*). Therefore, all of the population expansions could have occurred in the Late Pleistocene.

**Figure 3:**
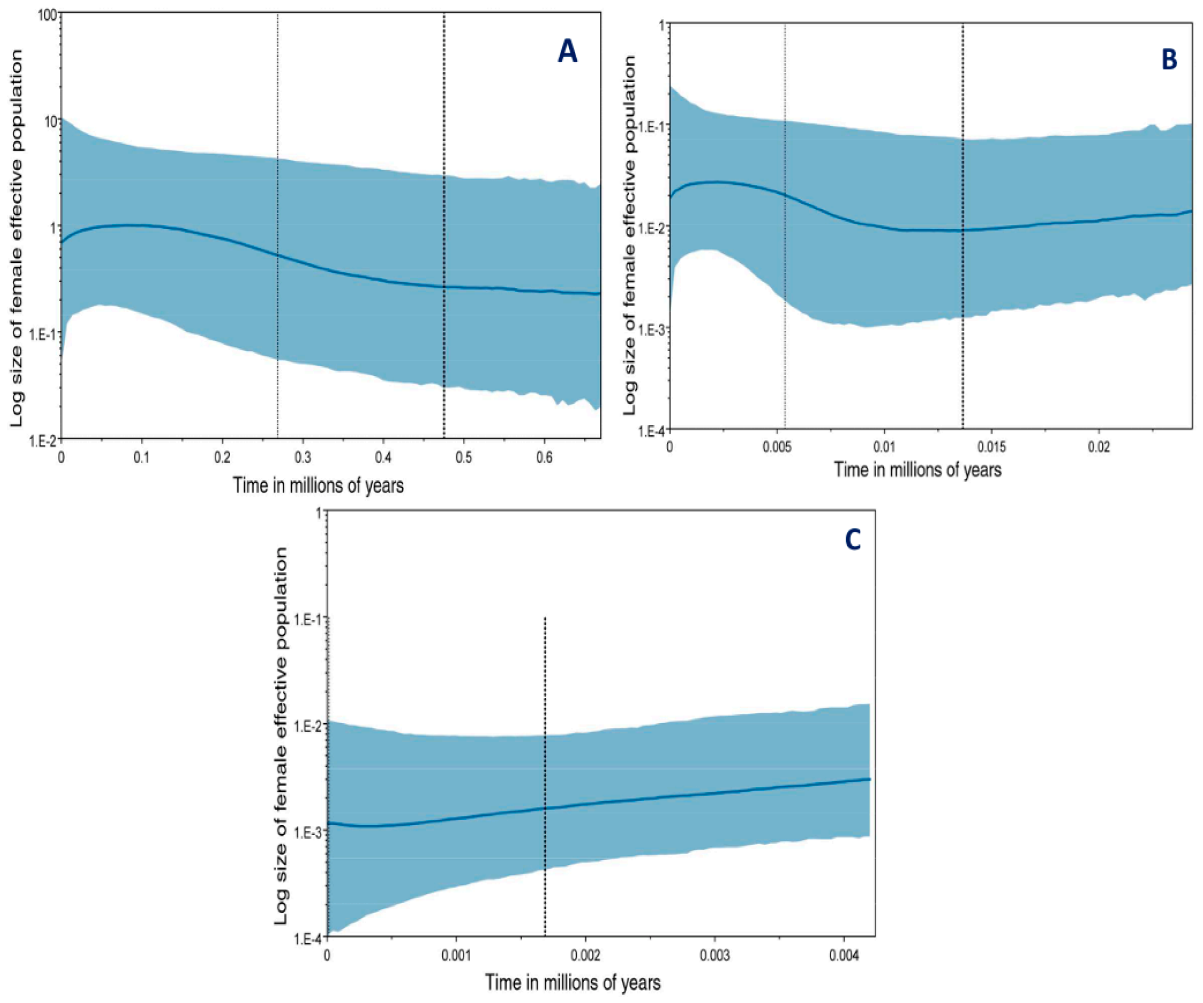
Bayesian skyline plot (BSP) analyses to determine the possible demographic changes across the natural history of *Galictis vittata*. (**A**) Using the mt*NADH5* gene for the last 600,000 years. (**B**) Using the mt*NADH5* gene for the last 30,000 years. (**C**) Using the *12S rRNA* gene for the last 4000 years. Some significant evidence of population expansion for *Galictis vittata* was found since 500,000 to 80,000 years ago, whilst evidence of population decrease was especially significant in the last 40,000 years ago.

For the mt*NADH5* gene, two BSP analyses were carried out. The first analysis covered the last 650,000 years (Figure 3A). From 500,000 years ago to approximately 250,000 years ago, there was steady, slow, and significant population growth (Bayes Factor = 6.3). From 250,000 years ago to 80,000 years ago, this population expansion of female lineages intensified (Bayes Factor = 8.1). Nonetheless, a population decline occurred in the last 40,000 years (Bayes Factor = 5.8). A second BSP analysis was performed exclusively for the last 30,000 years (Figure 3B). A slight but steady population decline was observed from 30,000 to 10,000 years ago (Bayes Factor = 5.5), but significant population expansion was observed from 10,000 to approximately 1000 years ago (Bayes Factor = 7.8), while significant population decline was again observed in the last thousand years (Bayes factor = 6.1). Therefore, there is significant evidence that the population of *G. vittata* has not remained constant over the last 650,000 years. The BSP analysis using the mt*12S rRNA* gene (Figure 3C) did not provide as much information as the previous analysis. It only allowed for the analysis of the last 4000 years, during which a gradual and progressive population decline of the female lineages in *G. vittata* was observed (Bayes factor = 5.7).

### 3.2. Phylogenetic Trees, Haplotype Networks, and Divergence Times for the Three Neotropical Ictonychinae Species and Other Neotropical Mustelid Genera

#### 3.2.1. Nucleotide Substitution Models

The best nucleotide substitution model for the mt*NADH5* gene was TN93 + I with BIC (=2761.50) and with AICc (=2338.73), while with lnL (=−1104.54) it was GTR + G + I. In the case of the mt*12S rRNA* gene, the best model using BIC (=1889.37) was T92 + G, while with AICc (=1608.91) and with lnL (=−760.33) the best models were TN93 + G and GTR + G + I, respectively. The best nucleotide substitution models for the mt*Dloop* marker were T92 + G for BIC (=1443.95), HKY + G for AICc (=1265.62), and GTR + G + I for lnL (=−595.84). The most optimal models were used in constructing the ML and BI trees.

#### 3.2.2. Maximum Likelihood, Bayesian Inference, and DensiTree Trees

**Figure 4:**
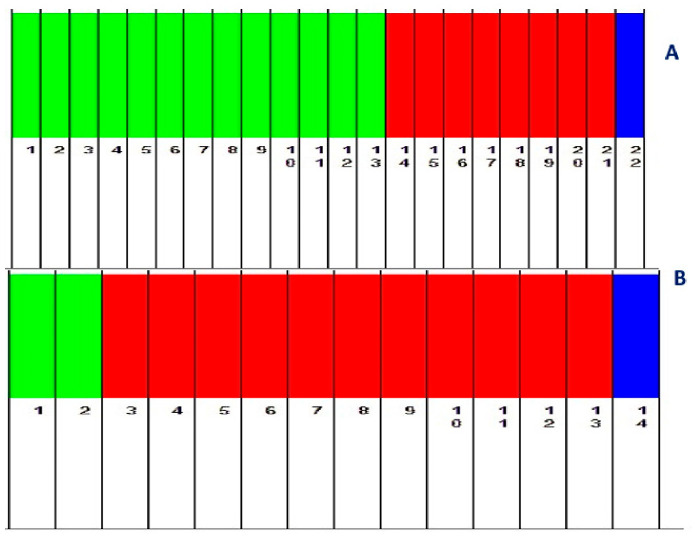
Genetic differentiation of three Neotropical Ictonychinae species by means of BAPS. Each different color indicates a different species. (**A**) Using the mt*NADH5* gene, three different species were differentiated. Green: 1–13 = *Galictis vittata*; red: 14–21 = *Galictis cuja*; blue: 22 = *Lyncodon patagonicus*. (**B**) Using the mt*12S rRNA* gene, three different species were differentiated. Green: 1–2 = *Galictis cuja*; red: 3–13 = *Galictis vittata*; blue: 14 = *Lyncodon patagonicus*. Therefore, both mitochondrial genes perfectly differentiated the three extant Neotropical Ictonychinae species.

All of the phylogenetic trees obtained perfectly differentiated the three Neotropical species of Lyncodontini. In fact, a previous BAPS analysis with the mt*NADH5* and mt*12S rRNA* genes also differentiated them (three species, Log (marginal likelihood) of optimal partition = −361.80, *p* = 1.0, for the first gene; three species, Log (marginal likelihood) of optimal partition = −61.98, *p* = 0.991, for the second gene) (Figure 4). The Kimura 2P genetic distances among the different mustelid species for the three mt markers used can be seen in Table 7. In the case of the mt*NADH5* gene, the greatest distance among the three Lyncodontini species was found between *G. cuja* and *G. vittata* (Da = 18.7%) and the lowest between *L. patagonicus* and *G. cuja* (Da = 16.0%). For the mt*12S rRNA* gene, the genetic distances were considerably smaller. The greatest distance among the three Neotropical Ictonychinae species was estimated between *G. cuja* and *L. patagonicus* (Da = 2.4%) and the smallest between *G. cuja* and *G. vittata* (Da = 1.8%). Finally, the mt*Dloop* marker showed the greatest distance between *L. patagonicus* and *G. vittata* (Da = 10.3%) and the smallest between *G. vittata* and *G. cuja* (Da = 6.3%). Therefore, the sequences that most differentiated between the three species of Lyncodontini and the remaining mustelid taxa used the mt*NADH5* gene.

**Figure 5:**
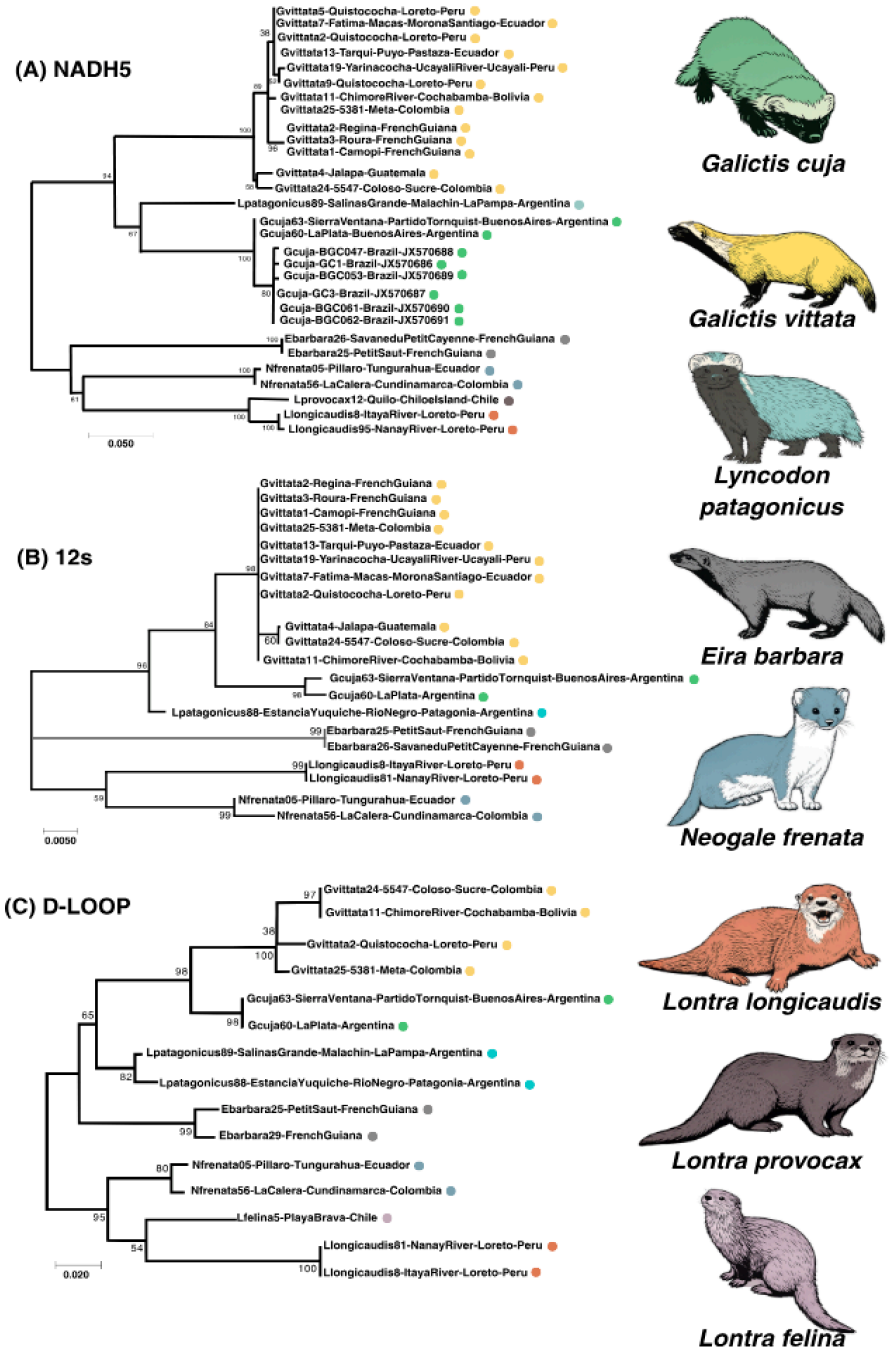
Maximum likelihood (ML) trees for different genera and species of Neotropical mustelids. (**A**) Using the mt*NADH5* gene with 29 specimens of 7 different species: 13 specimens of *Galictis vittata*, 8 specimens of *Galictis cuja* (specimens with numerical codes; their sequences were obtained from GenBank), 1 specimen of *Lyncodon patagonicus*, 2 specimens of *Neogale frenata*, 2 specimens of *Lontra longicaudis*, 1 specimen of *Lontra provocax*, and 2 specimens of *Eira barbara*. (**B**) Using the mt*12S rRNA* gene with 20 specimens of six different species: 11 specimens of *Galictis vittata*, 2 specimens of *Galictis cuja*, 1 specimen of *Lyncodon patagonicus*, 2 specimens of *Neogale frenata*, 2 specimens of *Lontra longicaudis*, and 2 specimens of *Eira barbara*. (**C**) Using the mt*Dloop* marker with 15 specimens of seven different species: 4 specimens of *Galictis vittata*, 2 specimens of *Galictis cuja*, 2 specimens of *Lyncodon patagonicus*, 2 specimens of *Neogale frenata*, 2 specimens of *Lontra longicaudis*, 1 specimen of *Lontra felina*, and 2 specimens of *Eira barbara*. In nodes, bootstrap percentages.

**Figure 6:**
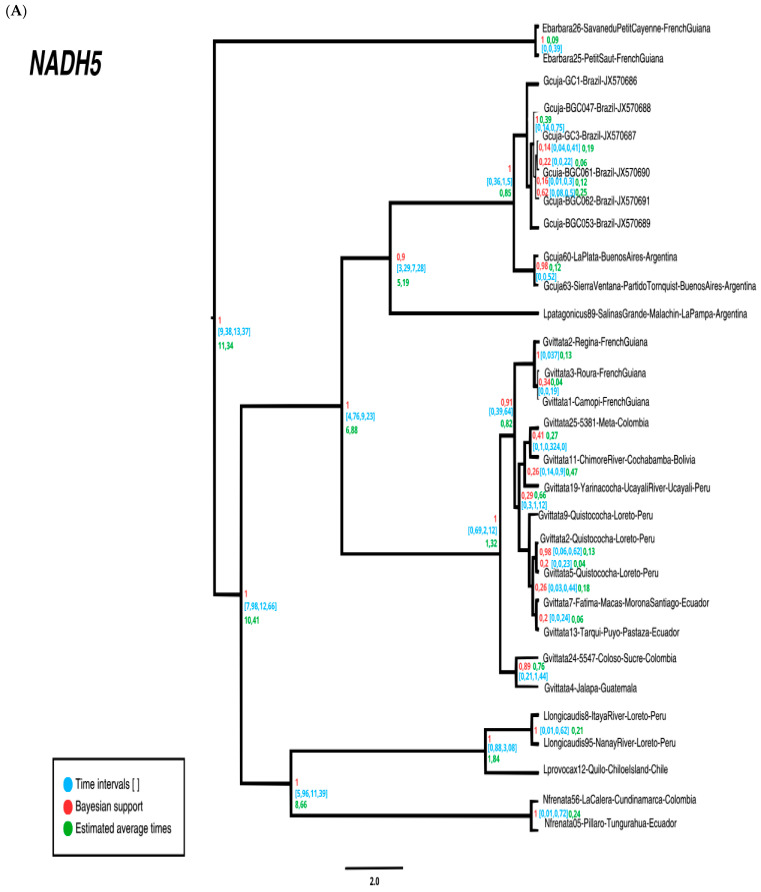

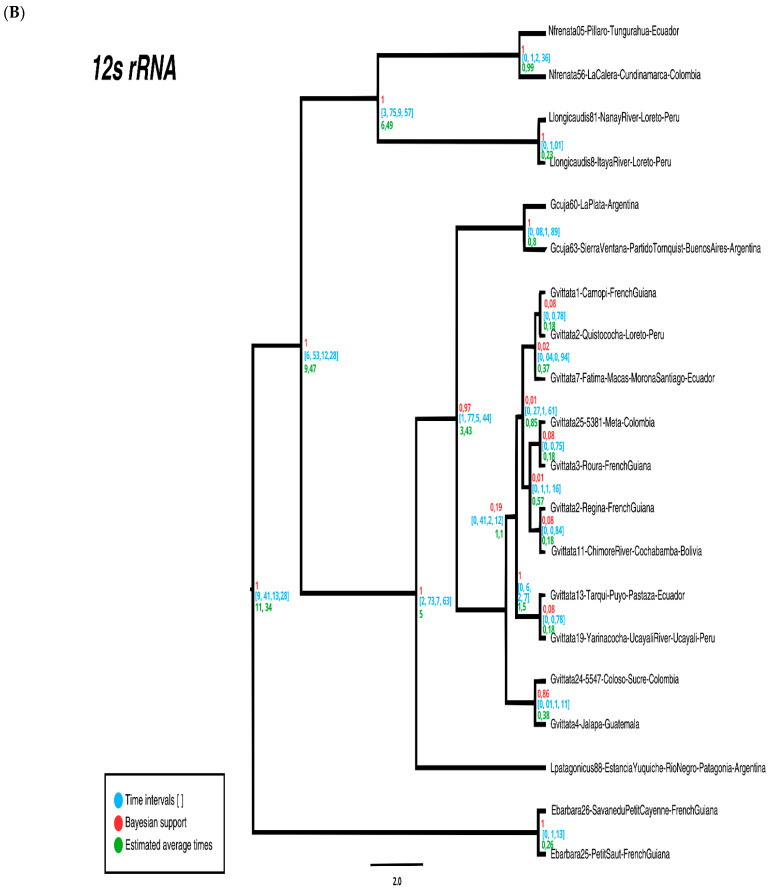

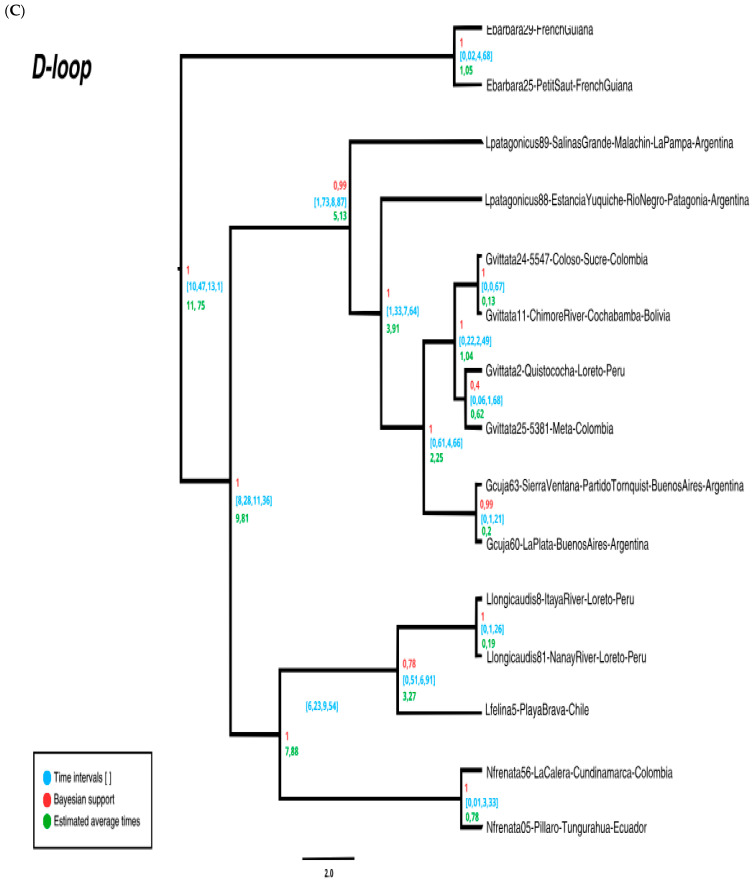
Bayesian inference (BI) trees for different genera and species of Neotropical mustelids. (**A**) Using the mt*NADH5* gene with 29 specimens of seven different species: 13 specimens of *Galictis vittata*, 8 specimens of *Galictis cuja* (specimens with numerical codes; their sequences were obtained from GenBank), 1 specimen of *Lyncodon patagonicus*, 2 specimens of *Neogale frenata*, 2 specimens of *Lontra longicaudis*, 1 specimen of *Lontra provocax*, and 2 specimens of *Eira barbara*. (**B**) Using the mt*12S rRNA* gene with 20 specimens of six different species: 11 specimens of *Galictis vittata*, 2 specimens of *Galictis cuja*, 1 specimen of *Lyncodon patagonicus*, 2 specimens of *Neogale frenata*, 2 specimens of *Lontra longicaudis*, and 2 specimens of *Eira barbara*. (**C**) Using the mt*Dloop* marker with 15 specimens of seven different species: 4 specimens of *Galictis vittata*, 2 specimens of *Galictis cuja*, 2 specimens of *Lyncodon patagonicus*, 2 specimens of *Neogale frenata*, 2 specimens of *Lontra longicaudis*, 1 specimen of *Lontra felina*, and 2 specimens of *Eira barbara*. In nodes, posterior probabilities (red) and temporal split estimations (green) (and into parentheses, light blue, 95% high posterior density, HPD, as confidence intervals; values in millions of years).

**Figure 7:**
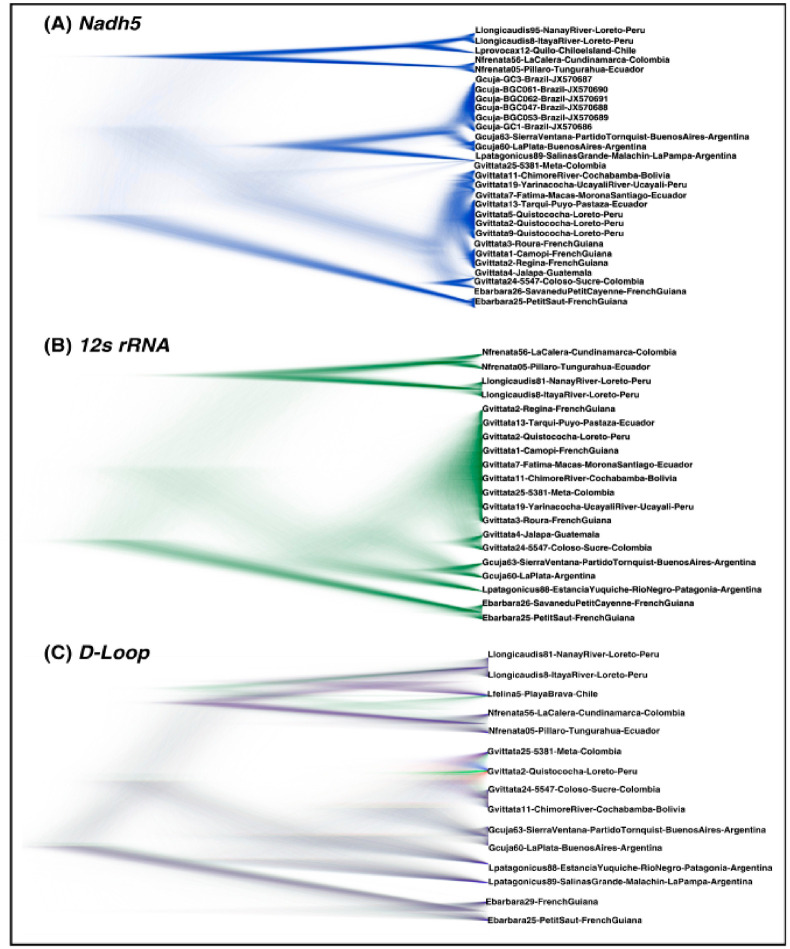
DensiTree (DT) consensus tree using the angled procedure for different genera and species of Neotropical mustelids. (**A**) Using the mt*NADH5* gene with 29 specimens of seven different species: 13 specimens of *Galictis vittata*, 8 specimens of *Galictis cuja* (specimens with numerical codes; their sequences were obtained from GenBank), 1 specimen of *Lyncodon patagonicus*, 2 specimens of *Neogale frenata*, 2 specimens of *Lontra longicaudis*, 1 specimen of *Lontra provocax*, and 2 specimens of *Eira barbara*. (**B**) Using the mt*12S rRNA* gene with 20 specimens of six different species: 11 specimens of *Galictis vittata*, 2 specimens of *Galictis cuja*, 1 specimen of *Lyncodon patagonicus*, 2 specimens of *Neogale frenata*, 2 specimens of *Lontra longicaudis*, and 2 specimens of *Eira barbara*. (**C**) Using the mt*Dloop* marker with 15 specimens of seven different species: 4 specimens of *Galictis vittata*, 2 specimens of *Galictis cuja*, 2 specimens of *Lyncodon patagonicus*, 2 specimens of *Neogale frenata*, 2 specimens of *Lontra longicaudis*, 1 specimen of *Lontra felina*, and 2 specimens of *Eira barbara*.

All of the phylogenetic trees obtained showed that the oldest branch to diverge is the one corresponding to the current *E. barbara*. In the ML trees (Figure 5), the position of this branch varies depending on the gene analyzed. Using the m*tNADH5* gene, the branch that gives rise to *E. barbara* is more closely related to the clade containing *N. frenata* and two otter species (*L. longicaudis* and *L. provocax*) than the clade containing the three Lyncodontini species. Using the mt*12S rRNA* gene, the *E. barbara* branch forms a tritomy that links it equally to the Neotropical Ictonychinae clade and the Neotropical Mustelinae (*N. frenata*) and Lutrinae (*L. longicaudis*) clade, while with the mt*Dloop* marker, the branch giving rise to *E. barbara,* is more closely related to the Lyncodontini than the clade formed by Mustelinae (*N. frenata*) and Lutrinae (*L. longicaudis* and *L. felina*). The three BI trees clearly show that the *E. barbara* branch is the first to diverge (*pp* = 1 for all three mt markers) (Figure 6). Similarly, the DT trees (Figure 7) for the three mt markers considered show that the densest and longest and therefore oldest branch is the one corresponding to *E. barbara*. Therefore, the BI and DT trees fully coincide with what was reported by Koepfli et al. [10].

All phylogenetic trees also showed, consistent with the findings of Koepfli et al. [10], a stronger relationship between Mustelinae and Lutrinae than between either of these taxa and the three Lyncodontini species or with *E. barbara*. For the ML trees with the mt*NADH5* and mt*12S rRNA* genes, the bootstrap percentages were not particularly high for the association of Mustelinae and Lutrinae (61 and 59%, respectively), but for the mt*Dloop* marker (95%) with the ML tree and for the three BI trees (*pp* = 1), this association was highly significant. Similarly, the three DT trees showed dense and long branches that are associated with Mustelinae and Lutrinae; these branches being much denser and longer than those of the Lyncodontini species, which indicates that the evolution of these Neotropical Mustelinae and Lutrinae was not as long as that of *E. barbara* but is considerably older than that of the three Lyncodontini species.

Regarding the three species of Lyncodontini, some very interesting new genetic results are presented. ML and BI trees for the mt*NADH5* gene showed a stronger relationship between *L. patagonicus* and *G. cuja* than between the latter and *G. vittata* (67% and *pp* = 0.9). Similarly, DT trees for two of these genes (mt*NADH5* and mt*12S rRNA*) showed a stronger association between *L. patagonicus* and *G. cuja* than between *G. cuja* and *G. vittata*. The branches corresponding to *L. patagonicus* were consistently the densest and longest of the three Lyncodontini species, indicating that the ancestor of *L. patagonicus* gave rise to the ancestors of the two *Galictis* species. This same finding of a stronger phylogenetic relationship between *L. patagonicus* and *G. cuja* will also be discussed later in relation to NJ networks. Notwithstanding, the ML trees with the mt*12S rRNA* and mt*Dloop* markers (96% and 65%, respectively) and the BI trees, with the same genes, showed a stronger association between *G. vittata* and *G. cuja* (*pp* = 1 in both cases). The DT tree, with the mt*Dloop* marker, did not show a clear association between *L. patagonicus* and *G. cuja*, but the former clearly possessed the densest and longest branches (indicating greater age) of the three Lyncodontini species. Koepfli et al. [10] did not include any *L. patagonicus* sample in their study, but Sato et al. [12], who have been, to date, the only ones to include a specimen of *L. patagonicus* in their phylogenetic analyses with mustelids, also found that the ancestor of *L. patagonicus* was the first to diverge within the present Neotropical Ictonychinae, as we report here with small fragments of mtDNA.

Within *G. vittata*, the findings shown above (genetic heterogeneity statistics and BAPS analyses) are corroborated by the phylogenetic trees. ML and BI trees with the mt*NADH5* gene detect three clusters within *G. vittata*. The most divergent cluster corresponds to the Guatemalan specimen and the specimen from northern Colombia (Sucre department) (100% and *pp* = 1, respectively). Subsequently, a strictly South American cluster appears in these two trees (82% and *pp* = 0.91, respectively), which is further divided into two sub-clusters. One contains the three specimens from French Guiana (96% and *pp* = 1, respectively), and the other contains the remaining sequences from specimens sampled in the Colombian Eastern Llanos, various locations in the Ecuadorian Amazon, different localities in the Peruvian Amazon, and one from the Chimoré River in Cochabamba, Bolivia. Therefore, three genetically distinct groups are found within *G. vittata* in the analysis using the largest number of samples of this species here analyzed. The DT tree with this same marker clearly shows the existence of the first group (Central America–northern Colombia) and the second group (French Guiana), but it also differentiates two smaller groupings within the third group detected by the previously discussed phylogenetic trees. On the one hand, a small grouping is distinguished, consisting of the specimen from the Colombian Eastern Llanos (Meta department), the specimen of Bolivian origin, and a specimen from the Peruvian Amazon (Ucayali department). On the other hand, the remaining sampled specimens appear in another small grouping, originating from the Peruvian and Ecuadorian Amazon. All three DT trees are consistent in showing that *G. vittata* is the taxon with the least dense and shortest branches, which could be consistent with it being the taxon with a more recent ancestor than *L. patagonicus* and *G. cuja*. All of the trees containing the most *G. vittata* sequences (mt*NADH5* and mt*12S rRNA*) are also consistent in showing that the divergence of the Central American–northern Colombian group is somewhat older than the diversification of the remaining *G. vittata* sequences. Therefore, there is strong evidence that the ancestor of *G. vittata* is the most recent of all of the extant Neotropical Ictonychinae species. The ML, BI, and BT trees for the mt*12S rRNA* gene (with a smaller number of sequences for *G. vittata* than with the previous marker) clearly detect only two clusters within this species. On the one hand, the first clade to diverge corresponds to the previously mentioned one from Central America–northern Colombia (60% and *pp* = 1, respectively). The second clade comprises all of the South American samples used, with those from French Guiana not distinguishable from the others, unlike what is observed with the mt*NADH5* gene. The smaller sample size for the mt*12S rRNA* gene and its lower rate of evolution compared to the mt*NADH5* gene may support this conclusion.

Regarding *G. cuja*, due to the small number of samples analyzed, we can offer few new results. In the case of the mt*NADH5* gene (the only one in which we were able to introduce sequences obtained from GenBank originating in Brazil), both sequences of Argentinian origin consistently showed an ancestor that diverged earlier than the sequences of Brazilian origin. This is visible in all three trees (ML, BI, DT) with the mt*NADH5* gene (and is also observable in the corresponding NJ network). One observable fact in some analyses (ML tree with mt*Dloop*, the three DT trees, and the NJ networks) is that the ancestor of *G. cuja* appears to have evolved earlier than the ancestor of *G. vittata*.

#### 3.2.3. Median Joining Networks and Temporal Divergences Between Neotropical Ictonychine Species and Between Other Genera of Neotropical Mustelids

The time divergence estimates from the BI trees closely matched those obtained by Koepfli et al. [10]. Using as a prior the value obtained for the origin of the branch that gave rise to *E. barbara* by Koepfli et al. [10] (11.8 mya, this being therefore the starting age of the root of all of our trees), the most important time divergence estimates can be seen in Table 8.

Three points are important to emphasize regarding these temporal divergence estimates between genera of Neotropical mustelids and within the Lyncodontini species observed in Table 8. First, the BI trees with the three small mt marker fragments studied showed relatively similar temporal divergence estimates among them, which emphasizes the importance of knowing the specific nucleotide substitution patterns of each gene or marker, as well as the mutation rates of each of the markers involved, for constructing the BI trees. Second, these temporal divergence estimates with the three mt gene fragments used (836 bp) were practically identical to those found by Koepfli et al. [8,10] with 22 different genes (21 nuclear and one mitochondrial, 12,000 bp). Third, Koepfli et al. [10] did not use any *L. patagonicus* sequence. Nevertheless, Sato et al. [12] did include a sample of *L. patagonicus* in their phylogenetic analysis. These authors estimated a temporal divergence between the ancestors of *G. vittata* and *G. cuja* of 2.03 mya (95% HPD: 1.34–2.86 mya; Multidivtime analysis) or 1.67 mya (95% HPD: 1.17–2.17 mya; BEAST analysis). Some of the estimates in this work and by Koepfli et al. [10] fall within the range of Sato et al.’s estimates. This is the case for the lower end of the interval calculated for the mt*12S rRNA* gene with BI, the average value for mt*12S rRNA* with MJ network, the average value and lower end of the interval calculated for BI for the *D-loop* marker, the average value of the MJ network for the *D-loop* marker, and the average value and lower end of the interval calculated by Koepfli et al. [10]. Notwithstanding, the average value of the MJ network for the mt*NADH5* gene, the average value and upper end of the BI interval for the mt*12S rRNA* gene, the upper end of the BI interval for the *D-loop* marker, and the upper end of the interval estimated by Koepfli et al. [10] were significantly higher than those estimated by Sato et al. [12]. The temporal divergence estimates obtained by Sato et al. [12] between *L. patagonicus* and *Galictis* were 2.89 mya (95% HPD: 2.42–3.76 mya; Multidivtime analysis) or 2.61 mya (95% HPD: 2.42–2.88 mya; BEAST analysis). Similarly to the previous case, some temporal split estimates between *Lyncodon* and *Galictis* were significantly higher in this study compared to the estimates made by Sato et al. [12]. This was the case for the average value and the upper end of the interval found for the mt*12S rRNA* gene using BI and the average value and the upper end of the interval found for the *D-loop* marker using BI. This result may have important implications for reconstructing the evolutionary history of these Neotropical mustelids.

**Figure 8:**
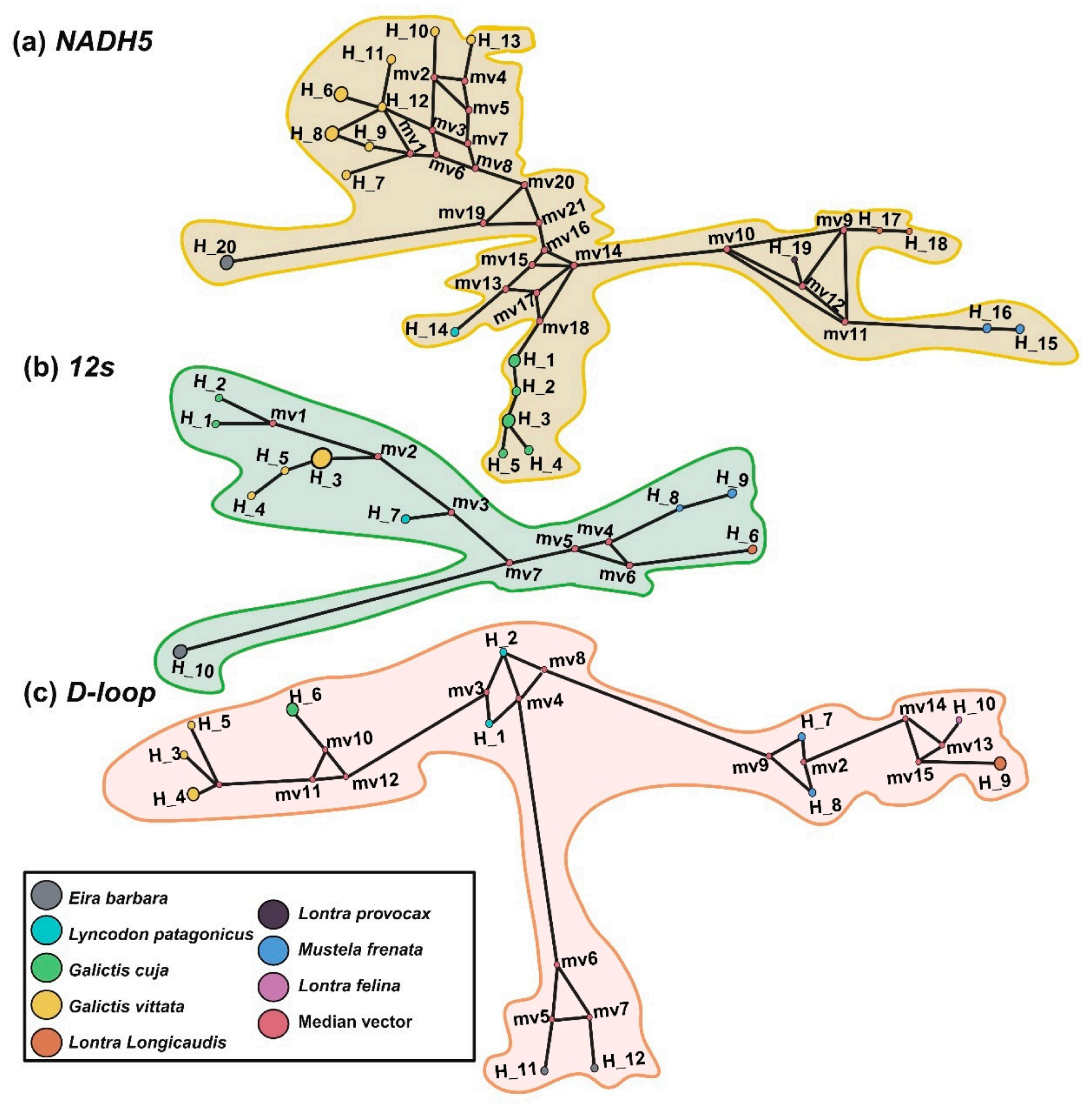
Median Joining (MJ) network containing the haplotypes of different genera and species of Neotropical mustelids. (**a**) Using the mt*NADH5* gene, with 29 specimens of seven different species: one haplotype of *Eira barbara* (gray), one haplotype of *Lyncodon patagonicus* (light blue), five haplotypes of *Galictis cuja* (green), eight haplotypes of *Galictis vittata* (yellow), two haplotypes of *Neogale frenata* (blue), two haplotypes of *Lontra longicaudis* (orange), and one haplotype of *Lontra provocax* (black). (**b**) Using the mt*12S rRNA* gene, with 20 specimens of six different species: one haplotype of *Eira barbara* (gray), one haplotype of *Lyncodon patagonicus* (light blue), two haplotypes of *Galictis cuja* (green), three haplotypes of *Galictis vittata* (yellow), two haplotypes of *Neogale frenata* (blue), and two haplotypes of *Lontra longicaudis* (orange). (**c**) Using the mt*Dloop* marker with 15 specimens of seven different species: two haplotypes of *Eira barbara* (gray), two haplotypes of *Lyncodon patagonicus* (light blue), one haplotype of *Galictis cuja* (green), three haplotypes of *Galictis vittata* (yellow), two haplotypes of *Neogale frenata* (blue), one haplotype of *Lontra longicaudis* (orange), and one haplotype of *Lontra felina* (pink). Red circles indicate undetected or extinct haplotypes. The size of the circles is proportional to the sample size of each haplotype.

MJ networks using haplotypes reinforced some of the results obtained, especially with the BI trees (Figure 8). These haplotype networks obtained from the markers mt*NADH5* (Figure 8a), mt*12S rRNA* (Figure 8b), and mt*D-Loop* (Figure 8c) showed consistent patterns of relatedness and divergence among the analyzed taxa. In all three cases, the *E. barbara* haplotype(s) were located near the origin of the network, suggesting a basal position within the studied assemblage, as assumed in the obtained phylogenetic trees. If we take the *E. barbara* haplotype as the most ancestral in the three MJ networks, the first haplotype to have originated from it was that of *L. patagonicus* (H14 in MJ network with mt*NADH5*, H7 in MJ network with mt*12S rRNA*, and H1 in MJ network with mt*Dloop*), and the divergence time between them was estimated at 5.87 ± 0.36 million of years (my) for mt*NADH5*, 6.08 ± 1.22 my for mt*12S rRNA*, and 7.19 ± 0.55 my for mt*Dloop*. The time elapsed between the haplotype of *E. barbara* and those of *G. cuja* (9.49 ± 0.64 my for mt*NADH5*, 8.6 ± 1.7 my for mt*12S rRNA*, and 9.17 ± 1.04 my for mt*Dloop*) and those of *G. vittata* (9.69 ± 0.21 my for mt*NADH5*, 10.81 ± 0.69 my for mt*12S rRNA*, and 10.24 ± 1.28 my for mt*Dloop*) showed that for the three mt markers considered, the time elapsed between the most ancestral haplotype was always greater for *G. vittata* than for *G. cuja* and both clearly longer than that found between the ancestral haplotype represented by *E. barbara* and the haplotypes of *L. patagonicus*. This suggests that the ancestor of *L. patagonicus* is much older than that of the other two Neotropical grisons. Moreover, the haplotype of *L. patagonicus* is temporally closer to that of *G. cuja* (3.20 ± 0.36 my for mt*NADH5*, 3.09 ± 0.34 my for mt*12S rRNA*, and 0.96 ± 0.14 my for mt*Dloop*) than that of *G. vittata* (5.57 ± 0.54 my for mt*NADH5*, 3.44 ± 0.81 my for mt*12S rRNA*, and 2.73 ± 0.37 my for mt*Dloop*). Thus, for all three markers, the time elapsed between the appearance of the *L. patagonicus* haplotypes was always shorter with respect to those of *G. cuja* than with respect to those of *G. vittata*. These results suggest that the evolution of the haplotypes of the three Neotropical Lyncodontini species occurred from south to north (with the ancestor of *L. patagonicus* being the oldest and that of *G. vittata* the most recent, as also observed in the DT trees). Within *G. vittata*, the split between the haplotypes of the Central American–northern Colombia group and those of the remaining South American group was estimated to have occurred at 0.67 ± 0.23 mya for mt*NADH5* and at 0.43 ± 0.26 mya for mt*12S rRNA*. Within *G. cuja*, the haplotype of the Argentine specimens appears to have given rise to the haplotypes of the specimens from southern and southeastern Brazil, as observed in the phylogenetic trees for mt*NADH5*. The most derived lineages correspond to otters and weasels, as they are further removed from the origin of the network. It can be observed that the temporal divergence times between the different mustelid taxa studied are relatively similar between the estimates obtained with BI trees and those obtained with MJ networks.

In short, the results obtained from these divergence times between the ancestors of different mustelid species using the three small fragments of the mt markers used, which were compared with the temporal estimates obtained by Koepfli et al. [10], turned out to be extremely similar. A good fit between the estimates from both studies would be very important because it would show that with samples offering DNA of low quality and quantity, and by sequencing very small mtDNA fragments, very robust results can be obtained both in the topologies of phylogenetic trees and in the temporal divergence estimates of the ancestors of extant taxa, at least in the case of mustelids.

## 4. Discussion

As Patterson [127,128] mentioned, many elementary aspects of the taxonomy and systematics of many mammal groups are still pending precise knowledge. In fact, even such basic aspects as the geographic distributions of many mammal species remain unknown [129], and this may affect their biological conservation to some degree [130].

This is the study that, to date, has analyzed the highest number of samples for some mitochondrial genes in some Lyncodontini, such as *G. vittata* and *L. patagonicus*. Although the sample sizes are small, they are the largest used for both species to date. In this sense, this work aims to make known to the scientific community the large gaps that exist in the taxonomic, systematic, and evolutionary knowledge of the extant Neotropical Lyncodontini species and aims to stimulate other research groups to obtain many more samples of these species for molecular genetic studies. A limitation of this study has to do with the very small DNA sampling and the fact that this sampling is only of mtDNA, which may make a rather limited contribution to the knowledge of supraspecific relationships in the extant Lyncodontini. However, given a relatively large sampling of *G. vittata* individuals and the focus on mtDNA, this study’s results are important at the infraspecific level for this species.

### 4.1. Genetic Diversity and Historical Demographic Changes in G. vittata

Analyzing genetic diversity in *G. vittata* yielded moderately high estimates of its levels, which is positive from a conservation perspective. It was important to use gene fragments that showed strong variability in their evolutionary rates, as this is crucial for a comprehensive understanding of mitochondrial DNA evolutionary dynamics. The mt*12S rRNA* marker showed the lowest nucleotide diversity and, in turn, had the lowest rate of evolution per million years (0.42%). The mt*NADH5* gene showed considerably higher nucleotide diversity (approximately 10 times higher than that of the previous gene), and its rate of evolution per million years (1.22%) was also three times higher. Finally, the m*tD-loop* marker showed very high nucleotide diversity (around 1.5 times higher than the nucleotide diversity found in the mt*NADH5* gene and about 14 times higher than the nucleotide diversity recorded for the mt*12S rRNA* gene), also being the marker with the highest evolution rate per million years of the three mt genes employed (4%, more than three times that of the mt*NADH5* gene and about 10 times that of the mt*12S rRNA* gene). Therefore, this study shows the importance of analyzing different mitochondrial genes (even if the fragments are small) with well-differentiated evolution rates to have a more accurate understanding of the overall evolution of mtDNA, as well as to be able to estimate with the greatest possible precision the evolution rates of these markers, which can potentially be used for other phylogenetically close organisms.

The average value for the three mt markers studied in *G. vittata* was H_d_ = 0.67 ± 0.13 and π = 1.03 ± 0.24 in %. These values were considerably higher than those found by Filippini [79] for *G. cuja* in Argentina (H_d_ = 0.09 and π = 0.15%, 21 specimens sequenced at the mt*NADH5* gene) but lower for H_d_ compared to two studies with *G. cuja* carried out in Brazil. Bornholdt [16], using 50 concatenated sequences of the mt*NADH5* and mt*Dloop* genes for *G. cuja* from southeastern (n = 9) and southern Brazil (n = 34) and Argentina (n = 7), estimated 17 different haplotypes with H_d_ = 0.92 ± 0.02, while Bontempo et al. [6], using the mt*Cyt-b* gene for 28 specimens from east–central Brazil, obtained H_d_ = 0.90, even though the range considered for *G. vittata* was notably larger in the present study (from Guatemala to Bolivia, passing through Colombia, Ecuador, Peru, and French Guiana). Nevertheless, the nucleotide diversity obtained in this study (π = 1.03%) for *G. vittata* was like that found for the Brazilian populations in those two studies conducted in Brazil (π = 0.85 ± 0.1% [16], π = 1.1% [6], respectively). Considering the area studied in the present research compared to that of the two studies conducted in Brazil (excluding the Argentinian samples), the mt genetic diversity of *G. vittata* may appear to be somewhat lower than that of *G. cuja*.

Using the marker analyzed with the largest number of *G. vittata* specimens, two procedures detected significant evidence of population expansion in female lineages. The mismatch distribution procedure detected the beginning of this population expansion between 370,000 and 635,000 ya. The BSP procedure fully coincided with these time estimates of population expansion for *G. vittata*. It detected slow but steady population expansion over the last 0.6–0.5 mya. Around 250,000 ya, this expansion intensified considerably until about 80,000 ya. This period coincides with the last phase of the Bonaerense [131], from 0.5 to 0.13 mya, and is characterized by a biozone of the giant sloth (†*Megatherium americanum*) and a large increase in mammals with Holartic origin in South America (deer and Muridae rodents, for instance). This was a time of stable climate, with a warm and humid environment. This probably caused an expansion of the maternal haplotypes of *G. vittata*. From that point, population decline began, becoming particularly pronounced from 40,000 ya onward, reaching its lowest point around 10,000 ya. During the cold period ca. 70,000 ya, large glaciers (Nevados) existed in Colombia and in northern South America [132]. It was estimated that the temperature was 7 °C lower than today in the Savanah of Bogotá [133]. From 70,000 to 35,000 ya [133], there was a period of intense expansion of glaciers. During that period, for instance, approximately 17,108 km^2^ of Colombia was covered by glaciers, and the ice reached down to 3000 ± 100 masl, with páramos at ca. 2500 masl. In fact, between 18,000 and 13,000 ya, the paramo that today is located between 3500 and 4500 masl was located at less than 2000 masl [134]. With the arrival of the Holocene (around 12,000 ya), around that time, new population growth began until about 1000 years ago. In fact, from 14,000 to 11,000 ya, precipitation and temperature increased and glacial retreat occurred. This allowed the formation of a lake (Tauca Lake; 43,000 km^2^) in the southern Bolivian Altiplano, which lasted until 11,000 ya [135]. This extremely cold period (11,000–10,000 ya) was named “El Abra” in the northern Andes, Younger Dryas, or Dryas III, in Scandinavia and northern Europe (12,900–11,700 ya) [136], or Tardi-glacial in central Europe [137], being crucial in the extinction of some large mammals, such as Gomphoteriidae (proboscideans), giant sloths, large notoungulates, and the famous *Smilodon*. Nonetheless, during a large part of the Holocene, there was an Optimum Climaticum [138,139], which occurred especially between 7000 and 3000 ya and was able to help, once again, population expansion of the female lineages of *G. vittata*. For instance, in the Savanah of Bogotá, the lake levels have increased in the last 9500–7000 years, and the Andean forests have expanded. At ca. 5000–4500 YA, the level of the Titicaca Lake increased, and, around the 10th century BCE, the temperature increased [135]. Although there were some periods of drought during the Holocene, these did not appear to negatively affect the size of *G. vittata* populations. Finally, a certain decrease in recent centuries has been detected in *G. vittata*. It is possible that this population reduction is the result of human hunting as well as climate change. The Little Ice Age (1400–1850 AC) is the most recent period of glacial advance. The ice sheets in Colombia descended on average to 4300 masl (currently, the ice front is ca. 5000 masl). Coinciding with the cooling of the environment, European colonizers arrived in the Andes. With their arrival came the introduction of domestic birds and mammals, as well as a considerable extension of maize and fruit crops in many Andean regions, in addition to habitat fragmentation and hunting the predators that could kill their domestic animals [140].

Bornholdt [16], in studying nine specimens of *G. cuja* from southeastern Brazil using mt*NADH5*, observed a pattern in the MJ network suggesting recent population expansion in southeastern Brazil, with all haplotypes from this Brazilian region differing from each other by a single mutational step and smoothly resembling a star-shaped structure. Similarly, with the concatenated sequences of mt*NADH5* and mt*Dloop*, the author found that the Fu’s F_s_ (= −2.55) and Tajima D (= −0.43) statistics were negative, which is consistent with population expansion, although only the former was statistically significant (*p* = 0.04). Conversely, there was no evidence of population expansion for the *G. cuja* population in southern Brazil (sample size of 34 specimens for the two previously mentioned mt markers). Furthermore, both statistical measures (Fu’s Fs and Tajima D) for that area of Brazil were positive (F_s_ = 4.82, and D = 1.9), which may be due to a bottleneck or the existence of several different gene pools in that area of Brazil. Nevertheless, Bornholdt [16] did not estimate the period or how long ago this population expansion occurred in southeastern Brazil for *G. cuja*.

Larger sample sets need to be analyzed to accurately determine how different Pleistocene events of a geological and climatological nature may have affected the population sizes of the three Lyncodontini species.

### 4.2. Molecular and Morphological Differentiation Between G. vittata and G. cuja and the Taxonomy of the Neotropical Lyncodontini Species

The molecular results obtained here showed that the three species of Lyncodontini (especially between *G. vittata* and *G. cuja*) can be perfectly differentiated, as previously determined by other authors [5,16], even with small fragments of mtDNA. For example, the genetic distances for the mt*NADH5* and *Dloop* genes showed high values among these three species (16–19% for mt*NADH5*, 6–11% for mt*Dloop*), which represents a considerable magnitude of genetic differences that allows for their perfect discrimination. Bornholdt et al. [5] had already detected considerable differences between the two *Galictis* species, around 12.7%, with mt*NADH5*. Conversely, the mt*12S rRNA* gene detected considerably smaller genetic differences, around 1.8–2.4%, which could be genetic distances between distinct populations or subspecies of a single species. Nonetheless, it should be noted that this latter gene has a considerably lower rate of evolution per million years than the other two genes used in this study. This means that some of the morphological characteristics traditionally used to differentiate these taxa of grisons are truly useful, as we noted in the Introduction. The present molecular study, like those of Bornholdt et al. [5,16], shows that the morphological characteristic used by Thomas [64] does have differential diagnostic value between both taxa.

Bornholdt et al. [5] also analyzed the sequences of 12 nuclear genes. All of them showed the same situation as the mitochondrial genes, even when analyzed individually. In no case did common alleles appear between the geographic regions corresponding to *G. vittata* and *G. cuja*; there was reciprocal monophyly among the alleles of the 12 nuclear genes studied. Separate analysis of these 12 nuclear segments showed that both species were differentiated by one mutation (*JAK1* and *MACF1* loci), two mutations (*TRHDE*, *AAMP2*, *ADORA3*, *PFKFB1*, and *PTPN4* loci), three mutations (*GNAT1*, *APOB*, and *RHO1* loci), and up to six different mutations (*RAG1* and *WT1* loci). With the 12 segments of these genes concatenated, strong differentiation between *G. vittata* and *G. cuja* was also observed. Taking into consideration both mitochondrial and nuclear markers, the separation support for both species was greater than 90%.

It is important to have morphological variables with a phylogenetic signal confirmed by molecular differentiation, as the distribution ranges of *G. vittata* and *G. cuja* overlap certain regions of South America. It is also important to highlight that the Bolivian *G. vittata* specimen that we analyzed in the present study came from the department of Cochabamba, where *G. cuja* has traditionally been reported. Therefore, it is important to be able to distinguish specimens of both species in sympatric areas in the field.

Our molecular analysis using the mt*NADH5* gene on 13 specimens of *G. vittata* (recall that this is the study with the most *G. vittata* samples and the largest geographical area analyzed to date using molecular markers for this species) detected three distinct geographic groups. One group consisted of two specimens, one from Central America (Guatemala) and the other from the northern Caribbean region of Colombia (Sucre department). The other group comprised specimens sampled from the rest of South America (the rest of Colombia, Ecuador, Peru, Bolivia, and French Guiana). Within this latter group, the mt*NADH5* marker identified another geographically consistent subgroup. The three specimens sampled in French Guiana formed their own cluster (the mt*12S rRNA* marker did not distinguish this last cluster). Therefore, there could be some correspondence between these three groupings and some of the subspecies of *G. vittata* that have traditionally been proposed by different zoologists. The first grouping could correspond well with *G. v. canaster*. Therefore, the morphological characteristics of this purported subspecies could have taxonomic value (dorsum is purer gray due to its light gray undercoat and guard hairs, with a light gray basal half, broad, black subterminal bands, and small white tips [141]). Furthermore, this subspecies would reach northern South America (at least northern Colombia). The problem with the two groupings found in South America would be one of taxonomic nomenclature. Schreber [142], with type locality in Suriname, defined the taxon that is now considered *G. v. vittata*. Notwithstanding, Bell [143], also using Suriname as the type locality, defined another grison species that would give rise to the so-called *G. allamandi*, which some authors still considered valid until recently [21,22], although most do not take it into account. On the other hand, Jensen and Tarifa [19] consider *G. allamandi* to be a synonym of *G. v. brasiliensis*, which is considered restricted to a significant portion of the Brazilian Atlantic coast. If *G. allamandi* is indeed a synonym of *G. v. brasiliensis*, then the form found in the geographic area of French Guiana could be *G. v. vittata*, and those analyzed in Colombia (excluding the one from the Sucre department), Ecuador, Peru, and Bolivia could be related to *G. v. andina*. Conversely, if *G. allamandi* is not a synonym of *G. v. brasiliensis*, then the French Guiana population could be called *G. v. allamandi*. For the remaining specimens, the possibility of naming them either *G. v. vittata* or *G. v. andina* should be considered. If *G. v. vittata* and/or *G. v. andina* were groupings that could be demonstrated with further molecular analysis involving a larger number of samples and genes, the characteristics of *G. v. vittata*, such as having dark brown or yellow-gray with white- or yellow-tipped hairs, or those of *G. v. andina*, such as having a dull yellowish stripe on the head and tips of yellowish dorsal hairs, would be diagnostic of those subspecies. We cannot make any inferences about *G. v. brasiliensis* because we have not analyzed any samples from the geographic range of that supposed subspecies of *G. v. vittata*.

Regarding the taxonomy of *G. cuja*, the research of Bornholdt [16], Bornholdt et al. [5], and Filippini [79], as well as the present work, make some interesting contributions from a taxonomic point of view. The Argentine haplotypes could represent the subspecies *G. c. huronax*, and the haplotypes from southern and southeastern Brazil could represent the subspecies *G. c. furax*. If subsequent studies corroborate the observations of the aforementioned initial studies, the morphological characteristics that could differentiate both putative subspecies and present a real morphological diagnostic feature are a buffy or ochraceous buff in the diagonal stripe and tips of dorsal hairs but a dark central portion of guard hairs that is dark gray and relatively short in *G. c. furax*, and very pale-buff or off-white haor in the diagonal stripe and tips of guard hairs and relatively more black in the central portion, giving the dorsum an even darker appearance in *G. c. huronax*. Because we do not have genetic results for other possible subspecies of *G. cuja* (*G. c. cuja* and *G. c. luteola*), we cannot make taxonomic inferences about them.

One comment regarding the phylogenetic trees concerns the BI and DT trees with the mt*Dloop* marker. This is the only marker used to analyze two specimens of *L. patagonicus*. In both analyses, the ancestor of the specimen from the Argentine province of La Pampa appears to have evolved earlier than the ancestor of the specimen from the Argentine province of Patagonia. The same is observed in the corresponding NJ network. This finding could suggest that the less southern form of *L. patagonicus* (*L. p. thomasi*) might be the original form and that the more southerly form might be a more derived one (*L. p. patagonicus)*. Moreover, this would coincide with Cabrera’s proposal [63] of the existence of two subspecies or groups within *L. patagonicus*. Nonetheless, molecular studies with many more samples of *L. patagonicus* should be carried out to confirm this hypothesis.

In most of the analyses performed (ML and BI trees with the mt*NADH5* gene, DT trees with the markers mt*NADH5* and mt*Dloop*, and in two out of three MJ networks with the mt*NADH5* and *Dloop* markers), a closer phylogenetic relationship was observed between *L. patagonicus* and *G. cuja* than between this last and *G. vittata*. Other analyses showed the more traditional view of both *Galictis* species being more closely related to each other than either of them to *Lyncodon* (ML and BI trees with the mt*12S rRNA* and mt*Dloop* markers, DT tree with *mtD-loop,* and the MJ network with mt*12S rRNA*). If we consider the first analyses mentioned, the taxonomy of *G. cuja* should show a closer relationship with *Lyncodon* than with *G. vittata*. Several alternative hypotheses could be presented. First, if more in-depth molecular studies incorporating a larger number of specimens, especially of *Lyncodon*, and a wide variety of different genes were to confirm this greater phylogenetic proximity between *L. patagonicus* and *G. cuja*, *G. cuja* would be renamed *Lyncodon cuja* (*Lyncodon* is older than *Grisonella*). Nevertheless, as a second possibility, because the genetic differentiation (at least with the mt*NADH5* gene) is of a high magnitude among the three species of Neotropical Ictonychinae (16–18%), perhaps each of them deserves to belong to a different genus: *Lyncodon patagonicus*, *Galictis vittata,* and *Grisonella* (Thomas 1921) *cuja*. Conversely, if we consider the results of the *12S rRNA* gene (1.8–2.4%), we could conclude that the current taxonomy that considers the existence of the two genera, *Galictis* and *Lyncodon*, is correct, or even that the three species could belong to a single genus (*Galictis*). *Galictis* (Bell 1826) would be chosen because this genus takes precedence over the genus *Lyncodon* (Gervais 1845). Therefore, a third species, *Galictis patagonicus*, would be added to the existing species of the genus *Galictis*, *G. vittata,* and *G. cuja*. Although this aspect discussed is speculative, only future research with a larger number of samples from the three species and with a wider variety of genetic markers will be able to more precisely elucidate the taxonomy of the Lyncodontini.

### 4.3. Tempo in Evolution of Neotropical Ictonychinae Species and in Other Genera of Mustelids

For the three mitochondrial markers studied (*NADH5*, *12S rRNA*, and *D-loop*), *E. barbara* was consistently recovered as the most basal lineage of the various mustelid genera studied, with divergence times around 9–13 mya, supporting its evolutionary distancing from the other Neotropical mustelids analyzed. The agreement in the phylogenetic position of this taxon and the proximity of the temporal divergence values of the branch that gave rise to this species among the three mitochondrial markers used reinforce the stability of this relationship, even though each gene showed different rates of evolution per million years. Even using very small fragments of several mitochondrial genes, this result fully agrees with Koepfli et al. [8,10], using a much more comprehensive set of genetic data. In the five most comprehensive studies on the molecular phylogeny of mustelids [8,10,11,12,15], there are some discrepancies regarding the group to which *E. barbara* belongs (Guloninae). Melinae and Guloninae were linked as sister groups in Koepfli et al. [10], although with weak statistical support. On the other hand, Sato et al. [12] found that Melinae was either a sister to Mellivorinae or constituted a distinct lineage. Nonetheless, Wolsan and Sato [11] detected that *E. barbara* was the outermost taxon of the Guloninae, which also coincided with Koepfli et al. [8,10]. Sato et al. [12] and Hassanin et al. [15] did not include any specimens of *E. barbara* in their respective studies. Some of these internal branches of the mustelid phylogeny are short, which indicates that mustelids underwent rapid diversification, with six of the eight subfamily clades splitting from one another within a span of about four million years, during the Middle to Late Miocene [10,12,144], as we also found in the present study.

The late Oligocene to early Miocene European *Plesictis* is the oldest known stem mustelid, and its oldest known species is *Plesictis plesictis*, with the oldest known occurrence of this species dating to 24.7–23.3 mya [145,146,147]. Kollias and Fernández-Morán [148] estimated the origin of Musteloidea to be approximately 32.4 to 30.9 mya in Asia. During the Miocene, fossils related to the subfamily containing *E. barbara* began to appear, which is consistent with the diversification of the mustelid subfamilies during the Miocene, as we observed for the origin of the branch that gave rise to *E. barbara*. During the period in which the Guloninae subfamily originated, there was a significant decrease in global temperatures. This cooling period coincides with the formation of a permanent Antarctic ice sheet in the Mid- to Late Miocene and an Arctic ice sheet in the Pliocene [149]. In addition, several major sea-level low stands occurred during the Late Miocene and Pliocene, including the Serravallian sea-lowering event near the beginning of the Late Miocene, 11–10 mya [150]. These changes in climate and sea level increased overall terrestrial aridity and seasonality, leading to the disappearance of tropical and subtropical forests and the emergence of a greater proportion of open vegetation habitats (woodlands and grasslands) [151]. By the early Late Miocene, fossil evidence indicates that the Eurasian continent was a mosaic of vegetation types and generally more heterogeneous in vegetation structure than in earlier periods. These changes in vegetation had a concomitant impact on faunal communities and may have caused diversification within mustelids due to geographic isolation, divergent selection among different habitats, and/or ecological opportunity through the creation of new niches. Changes in habitat and extinction of earlier lineages of mustelids may have created ecological opportunities that enabled the adaptive radiation of modern mustelids. The Guloninae, the branch that directly gave rise to *E. Barbara,* may have originated between 7.7 and 6.8 mya [10]. In a previous study of *E. barbara* [152], it was determined that the temporal differentiation between the population of southern Central America and northern Colombia and the remaining South American population occurred between 6.4 and 4 mya, coinciding with the end of the Miocene and the beginning of the Pliocene. This may indicate that the ancestor of *E. barbara* arrived in South America before the definitive closure of the Isthmus of Panama (3.5–2.8 mya), which shows that modern mustelids, like modern procyonids, arrived in South America much earlier than traditionally indicated from a paleontological perspective [126].

Another difference between the phylogenetic studies of Koepfli et al. [8,10], Wolsan and Sato [11], Sato et al. [12], and Hassanin et al. [15] refers to the position of the Mustelinae. Mustelinae was recovered as the sister group to the Lutrinae in Koepfli et al. [8,10], while Wolsan and Sato [11], Sato et al. [12], and Hassanin et al. [15] considered Ictonychinae and Lutrinae to be sister groups. This suggests that these clades diversified over a relatively short period of evolutionary time, and this can confound phylogenetic tree topologies because the phylogenetic signal from different DNA sequences is attenuated and often conflicts across such short branches. The results we present here agree perfectly with Koepfli et al. [8,10], as Mustelinae and Lutrinae were sister groups in all of the analyses we carried out. Our estimates of temporal genetic divergence between the ancestors of *Neogale* and *Lontra* ranged from 6 to 9 mya. These values were very similar to those of Koepfli et al. [8,10] obtained for the temporal divergence of Mustelinae and Lutrinae (8.7–9 mya) and somewhat lower than those obtained by Sato et al. [12] (8.9–12.5 mya) although similar to the lower end of the range estimated by Sato et al. [12] but much lower than the estimate made by Hassanin et al. [15] between Lutrinae and Mustelinae (16–18.3 mya).

Another major proliferation of mustelid taxa occurred during the Pliocene (5.3–2.8 mya). Genetic studies have revealed this strong second proliferation of mustelid taxa, which generated as many as 20 different lineages representing modern mustelid genera and species originating within a period of less than four million years [8,10]. This rapid lineage splitting is one of the hallmarks of adaptive radiation. For mustelids, this diversification was driven by the cooling of global temperatures, the restructuring and redistribution of biomes, and major faunal turnovers across different continents.

This second cycle of global cooling and drying on Earth during the Pliocene coincided with the onset of high-latitude glacial cycles [149], which resulted in a strong expansion of low-biomass vegetation, including grasslands and steppes at midlatitudes, and the development of taiga at high latitudes in Eurasia, North America, and probably South America [151]. Associated with this was a significant diversification of muroid rodents and passerine birds that exploited these new habitats, which in turn provided new niches for predators [153]. Many of the ancestors of modern *Martes* and *Mustela* species originated because of these processes, as they are specialists in hunting small rodents. King’s [154] hypothesis suggests that the evolution of small body size in *Mustela* (and, as we will argue later, also in the ancestor of *L. patagonicus*) was driven by adaptation to exploit abundant resources presented by rodent diversification during the Pliocene, especially rodents that conquered the subsoil and, with them, the mustelid predators that followed to prey on them. Koepfli et al. [8,10] note that the diversification of four of the five species within *Martes*, all closely associated with taiga forest habitat, coincides with the expansion of this habitat type across the Holarctic during the Plio-Pleistocene. This same phenomenon could have driven the beginning of the diversification of the Lyncodontini in South America, whose oldest extant genus may have been *Lyncodon*. This same process of adaptive radiation and diversification in mustelids during the Pliocene has also been detected in Felidae [155], and Cervidae [156] at very similar dates.

Koepfli et al. [8,10] supported Eurasia as the origin of Ictonychinae, while Sato et al. [12] favored either Asia (parsimony and ML trees) or Africa (BI tree). Where Ictonychini arose is also uncertain. Sato et al. [12] favored Asia (parsimony tree) or Africa (BI tree) or that the ancestral range extended on to both continents (ML tree), while the results of Koepfli et al. [10] are equivocal in this regard. The first occurrence of this clade was represented by the record of *Baranogale helbingi* from Podlesice, Poland [157,158,159,160]. This fossil site is the reference locality of the European Neogene mammal chronological unit MN 14 and has been dated as a biostratigraphic zone that spans 4.9–4.2 mya [161]. Recently, Marciszak et al. [162] dated to 3.6–3.2 mya the abundant *B. helbingi* material from one of the eight sites in Poland where this fossil species has been found. Like other European ichthyochini, *B. helbingi* disappeared during the early Pleistocene. Among the Eurasian fossil taxa of this subfamily, the first to be described were *Enhydrictis* and *Pannonictis* [163,164]. The first genus was recovered from the late Pleistocene deposits of Monte S. Giovanni (Sardinia) and has been named *Enhydrictis galictoides*. This form showed a strong affinity with the extant South American Lyncodontini *G. cuja* and *G. vittata*, even with the Neotropical Guloninae, *E. barbara* [163]. The second genus is well-characterized from the Plio-Pleistocene deposits of central and southern Europe. In fact, it is the best-known and most referenced genus in Eurasia [165].

Jiangzuo et al. [166] studied the rich Late Miocene assemblage of mustelids from northern and southern China. The authors differentiated different species, *Cernictis baskini*, *Cernictis lufengensis*, *Lutravus dianensis*, and *Shansictis xinzhouensis*. *Lutravus* (originally from Asia and also found in North America) and *Shansictis* (in a more basal position) were classified within Lyncodontini as the Neotropical *Galictis* and *Lyncodon*, whereas *Cernictis* was found as a basal member of Ictonychini (of Asian origin, but also present in North America). The divergence of the two tribes was likely to have occurred in the Late Miocene of eastern Asia, with the subfamily undergoing rapid intercontinental Asian American dispersals after its initial diversification.

According to the paleontological record, the forms most closely related to the current Neotropical Lyncodontini are the following fossil forms, the earliest originating from North America. *Trigonictis* arrived in the New World in the mid-Pliocene and was common in North American Blancan-age deposits [167], which are dated between 4.9 mya and 1.8 mya. *Trigonictis* differs little from the current *Galictis* [168], and two species have been distinguished by paleontologists: a larger *Trigonictis macrodon* and a smaller *Trigonictis cookii*. Another closely related genus and species of Ictonychinae is *Smithosinus bowleri*, which has strong affinity to *Trigonictis* and is possibly only a subgenus of the latter. In deposits located in Hagerman, Idaho, this representative of the North American Ictonychinae occurred in marshy habitats and for some paleontologists was possibly the ancestor of *G. cuja* [168,169]. Some authors suggest that *T. cookii* became larger and evolved into *T. macrodon* as a chronospecies [170]. Nevertheless, both *Trigonictis* species coexisted in several local faunas [168]. There has also been debate between the hypotheses that *Trigonictis* is either ancestral to *Galictis* [167] or congeneric with *Galictis* [171]. Just as some authors have concluded that *S. bowleri* may have been the ancestor of *G. cuja*, others have concluded that *T. macrodon* may have been the ancestor of *G. vittata*, whereas *T. cookii* was the ancestor of *G. cuja* [167]. Nonetheless, results using molecular genetic methods can offer a very different perspective on the evolution of Neotropical Lyncodontini. Additionally, the limb bones of *Trigonictis* are not as robust as those of *Galictis*, the M1 of *Trigonictis* is more like that of *Eira*, and *Trigonictis* is almost equally like *Eira* and *Galictis* [168]. *Galictis* fossils appeared in South America during the Marplatan land mammal age, Vorohuean subage (formerly Uquian land mammal age; 2.5 mya) [172,173,174]. Three taxa of the subgenus *Galictis* are known as fossils. *Galictis sorgentinii* occurred in the early Pleistocene of Argentina [172], *Galictis sanandresensis* from the San Andres Formation, Argentina, near where *G. sorgentinii* was found, and *G. vittata* is also known as a fossil [174]. Other fossil remains, such as *Galictis intermedius* (Pleistocene deposits in Minas Gerais, Brazil) and *Galictis allamandi* (=*vittata*) *fossilis* (Brazil), appear to be indistinguishable from the extant *G. vittata* [175]. With reference to *G. cuja*, fossils related to this extant species have also been found. This is the case for *Galictis hennigi* (subgenus *Grisonella*, from Argentina) [174], the extant *G. cuja* [20], and an unidentified *Galictis* mandible (Bolivia) [176]. Other forms, such as *Galictis major* and *Galictis robusta,* appear indistinguishable from *G. cuja* [174]. In fact, the aforementioned *G. hennigi* from Argentina [174] is known from a single specimen, and some authors consider it indistinguishable from *G. cuja*. The oldest fossil known of *Lyncodon* is a skull of *L. bosei* [177] from a site in the Ensenada Formation, Argentina [178]. This site is dated within a range of 1.07–0.99 mya [179]. Nevertheless, the oldest taxon of *Galictis* obtained in the South American fossil record is the aforementioned *G. sorgentinii* [180]. This fossil is referred to as the Vorohuean subage, and it was dated about 3.0–2.4 mya [180]. Nevertheless, it should not be forgotten that some authors have not been able to differentiate *G. sorgentinii* from *G. cuja*.

The study by Koepfli et al. [10] estimated the origin of Ictonychinae at 7.9–8.2 mya, while these figures were somewhat higher in the study by Sato et al. [12], reaching 9–9.5 mya. Our work could not provide estimates in this regard, as we did not study Ictonychinae from Asia or Africa. Notwithstanding, comparing our estimates of temporal divergence among Neotropical Lyncodontini taxa with those obtained by the two aforementioned studies do provide interesting insights into the evolution of this group of mustelids in Central and South America. The extreme values found for the divergence of the ancestors of *Lyncodon* and *Galictis* in [12] were between 2.4 and 3.8 mya ([10] did not include *Lyncodon* in its analysis). Our average time estimate was 4.8 mya, with extreme values between 2.7 and 6.9 mya, considerably higher than the previously discussed estimate [12]. That is, our estimates identified the divergence of *Lyncodon* and *Galictis* primarily during the early Pliocene. Nonetheless, 5 mya ago, the Isthmus of Panama had not yet definitively closed (2.8–3.5 mya, [181,182]), which creates a dilemma that we will attempt to resolve in the next section. The presumed time split between *G. vittata* and *G. cuja* ranges from 2.8–3.0 mya [10] to 1.2–2.9 mya [12]. Bornholdt et al. [5] estimated that the differentiation based on the nuclear genes they analyzed was consistent with the estimate of divergence time between *G. cuja* and *G. vittata*, around 2 to 3 mya. Our average estimate of the temporal split between *G. vittata* and *G. cuja* was 3.4 mya, with extreme values between 1.4 and 6.9 mya, slightly higher than previous temporal estimates but with considerable overlap. Therefore, there appears to be agreement between the temporal divergence estimates between *G. vittata* and *G. cuja* obtained in previous studies and the estimates presented here. The split between the two species would have occurred in the late Pliocene or early Pleistocene.

### 4.4. Mode of Evolution of Neotropical Ictonychinae Species

The traditional view of when and how the ancestors of present-day grisons arrived in South America is as follows [5,79]. The fossil record indicates that Lyncodontini probably originated in North America, descending from a common ancestor, the Pliocene genus *Trigonictis* [170]. Representatives of the genus *Galictis* then colonized South America during the Great American Biotic Interchange (GABI) [183] (the time of divergence among grisons was around 2.5–2.8 mya, during the second and largest wave of mustelid diversification in the Pliocene), through the complete emergence of the Isthmus of Panama [12,172], which provided a land bridge for the exchange of fauna between North and South America. The first fossil record of *Galictis* in South America appeared in Argentina in the Vorohuean subage of the late Pliocene [171,183,184], dated at 3.0–2.5 mya. Therefore, several North American ancestors of *Galictis* (paleontological view) or a single *Galictis* ancestor (molecular view) invaded South America via the Panamanian isthmus early during the GABI [183,184], soon afterwards giving rise to the two extant species, as well as additional extinct members of the genus [171]. The traditional interpretation that current mustelid taxa in South America are largely descended from North American species that arrived as part of the GABI following the rise of the Panamanian isthmus is repeated time and again [10,12]. Undoubtedly, there are cases that fit this interpretation well. For example, this relationship is clearly indicated for the clade of New World otters, in which *L. canadensis* is a sister to *L. felina* and *L. longicaudis*, with the latter two species found in the Neotropics. Their corresponding split is estimated to have occurred 2.8–3.4 mya (95% HPD: 1.6–5.2 mya), which overlaps well with the timing of the formation of the Panamanian land bridge [10]. Nevertheless, the case of the Neotropical Lyncodontini may have been very different.

Our new results could allow us to hypothesize a different colonization route by the ancestors of present-day Neotropical Lyncodontini. All of the phylogenetic trees obtained, but especially the three haplotype networks (MJ networks), showed that, assuming the haplotypes of the ancestor of *E. barbara* were the first to diverge among the mustelids studied, the haplotypes of *L. patagonicus* were the first to appear, followed by those of *G. cuja*, and finally by those of *G. vittata* (in fact, in several analyses, the haplotypes of *G. cuja* are closer to those of *L. patagonicus* than those of *G. vittata*, with the taxonomic implications discussed above). Similarly, the DT trees showed, for the three mt markers, that the branches for *L. patagonicus* (first) and for *G. cuja* are denser and deeper than those for *G. vittata*, which would appear to be a more recent species. This would imply that the evolution and speciation of modern Neotropical Lyncodontini originated in southern South America (the Argentine Pampas and Patagonia, *L. patagonicus*), passing through north–central Argentina, southern and southeastern–central Brazil, Bolivia, and southern Peru (*G. cuja*), and more recently giving rise to *G. vittata* in what is now north–central Peru, Ecuador, Colombia, and Central America (with the populations in the latter area being the most recent). Furthermore, this process would have begun in South America at the end of the Miocene and the beginning of the Pliocene, well before the definitive closure of the Isthmus of Panama (2.8–3.5 mya). This hypothesis would significantly alter the traditional paleontological and molecular view on the matter. This hypothesis is based on the following points.

(1) The traditional viewpoint has been that the GABI occurred throughout the development of an overland corridor across the Panamanian region and that this ended around 3.0 mya. Nonetheless, it was already known by paleontologists that there was limited exchange of taxa in both directions prior to this time and prior to the development of a well-defined “land bridge,” with the precursors being designated as “New Island Hoppers” [185] or “heralds” [171,186]. Geological and tectonic reconstructions [187] have shown that the Panamanian region contained a series of islands around 6 mya, with the Central American Seaway largely interrupted by an evolving volcanic arc about 12 mya as the southwestern margin of the Caribbean Plate collided with the South American continent. Apparently, this fostered the entry of the first sloths into North America around 9 mya [188] and the arrival of the first Holartic mammals into South America (Argentina) approximately 7.3 mya during the Huayquerian (Late Miocene, 9–6.8 mya). They were procyonids of the genera *Cyonasua* and *Chapalmalania* [189,190]. In fact, four taxa of North American origin (proboscideans, tapirids, peccaries, and dromomerycins such as *Suramerix*), dating in 9.5 mya to the Amazon Basin from beds putatively older than those reported for both procyonids, have been found. The next immigrants were endemic genera of sigmodontine rodents and a procyonid genus *Parahyaenodon* from the Montehermosan Age (6.8–4 mya, Late Miocene–Early Pliocene; [191,192]). Many of these taxa were linked to forest environments, and they were not well-adapted for colonizing islands. Therefore, a terrestrial bridge was necessary for these colonization events. All of these events could be related to the development of the Baudo Bridge around 10 mya, which connected northwestern South America to the central area of Panama throughout the cordilleras of San Blas and Baudo [193]. They could also be linked to the closure of the Central American Sea Channel, which happened around 10 mya [194,195]. Recently, Wolsan and Sato [62] proposed a very interesting hypothesis. These authors showed that previously examined carnivoran species that include tetrapods in their diets possess functional *TAS1R1*–*TAS1R3* genes due to the presence of substances such as purine 5′-monophosphate ribonucleotides, which are the main agonists of *TAS1R1–TAS1R3* genes in carnivorans. Nonfunctional *TAS1R1–TAS1R3* genes have previously been found only in carnivoran species specialized in feeding on fish, aquatic mollusks, and aquatic invertebrates. This is expected, as purine 5′-monophosphate ribonucleotides are relatively scarce in tissues of living or recently dead fish and aquatic invertebrates. Wolsan and Sato [62] demonstrated that *L. patagonicus* and *G. cuja* are exceptional among carnivorans in that they possess a nonfunctional *TAS1R1–TAS1R3* genes despite their diets high in umami-taste-eliciting compounds, including purine 5′-monophosphate ribonucleotides. The results of the study indicated that the lack of *TAS1R1–TAS1R3* gene function in these Lyncodontini is a remnant of their common ancestor that lived within a time interval of around 3 to 9.5 mya as part of the Lyncodontini’s stem lineage. Because loss of taste receptor function is achieved through a stochastic process that continues over evolutionary time, Wolsan and Sato’s [62] hypothesis claimed a prolonged semi-aquatic episode in the evolutionary history of the Lyncodontini’s stem lineage. In fact, *Lutravus* (7.5 mya) [166] exhibits otter-like dentition suggestive of otter-like feeding habits and habitat (which would support the phylogenies of Wolsan and Sato [11], Sato et al. [12], and Hassanin et al. [15] versus the phylogeny of Koepfli et al. [8,10] in reference to the relationship between Ictonychinae and Lutrinae). If some of the ancestors of modern Lyncodontini maintained their aquatic habits, this would have greatly favored the colonization of South America by this group long before the definitive closure of the Isthmus of Panama. Therefore, either as “New Island Hoppers” or via the land bridges that existed during the late Miocene, the ancestors of present-day Neotropical Lyncodontini were able to reach South America before the definitive closure of the Isthmus of Panama (2.8–3.5 mya). This latter fact has greatly influenced the interpretation, even with molecular data, of when the ancestors of present-day Lyncodontini arrived in South America [5,12]. Just as with procyonids, such as *Nasua* and *Procyon*, which were thought to have entered South America during GABI 4 (0.125 mya [183]) and in recent years have been molecularly demonstrated to have developed in situ in northwestern South America well before the definitive closure of the Isthmus of Panama (10–13 mya) [158,196], something similar appears to have occurred with mustelids, and at least the evolution of Lyncodontini seems to have taken place in situ in South America from the late Miocene and early Pliocene. It had also been shown that the ancestors of the modern *E. barbara* may have arrived in South America between 6.4 and 4 mya [152]. Therefore, the arrival of the ancestors of present-day Neotropical Lyncodontini around 5–6 mya should not be viewed with surprise.

(2) The oldest surviving branch of the Neotropical Lyncodontini is that of *L. patagonicus*. This means that the earliest forms related to the current *L. patagonicus*, which is highly adapted to the semi-desert climate of what is now Patagonia, and with an increasing number of small, subterranean prey (especially rodents), adapted their morphology to the ecology of these rodents. Recall that many genera of sigmodontine rodents appear in the South American fossil record at that time (6.8–4 mya, Late Miocene–Early Pliocene; [192]). Just as in the Northern Hemisphere, with the formation of steppes (taiga, tundra) due to the cold, dry climate that caused much of the water on Earth to be frozen, allowing for the rapid and prolific adaptive radiation of *Martes* and *Mustela* (the latter with very small bodies for capturing prey underground) due to the proliferation of small mammals and birds [10], South America also underwent these events. For example, the first camelids in South America (represented by the endemic genus *Lama*) were recorded around 4.2–4.0 mya (Pliocene) [197]. If camelids well-adapted to the savanna environment already existed in South America at that time, it is because savannas already existed because of cooling and desertification of much of South America due to the planetary cooling that occurred at the end of the Miocene and during much of the Pliocene.

Our hypothesis is strongly supported by several morphological studies conducted by various authors. Law [198] showed that the expansion of grasslands and diversification of rodents and lagomorphs during the Middle to Late Miocene led to increased clade carrying capacity within extant mustelids, particularly Mustelinae, Lutrinae, and Ictonychinae. To reach these conclusions, Law [198] analyzed an important morphological dataset that incorporates cranial shape, body size, and body shape to understand phenotypic evolution in a clade of extant small-sized predators, finding that within extant mustelid sub-clades (particularly Ictonychinae, Mustelinae, and Lutrinae), evolutionary shifts towards more robust crania, small body sizes, and elongated bodies all occurred during the Mid-Miocene Climate Transition, around 15.97–11.61 mya, a period of time characterized by arid climates, open habitat expansions, and rodent and lagomorph diversification, as we previously explained. Shortening the rostrum and broadening the mastoid and zygomatic arch breadth are often associated with increases in relative bite forces. Therefore, an evolutionary shift towards these broader cranial shapes favoring larger jaw muscle attachment areas may counteract the weaker bite forces associated with smaller body sizes. Concurrent shifts towards smaller, more elongated body plans would enable these mustelids to actively chase prey down into burrows or crevices, as is the case with the extant *L. patagonicus*, and their relatively large bite forces for their smaller sizes would facilitate the successful dispatch of prey that can be up to 10 times larger than many mustelids.

During the Late Pleistocene and the Holocene, *L. patagonicus* occurred in eastern portions of Buenos Aires province, where it is now absent [56]. This geographic occurrence was related to the existence of more arid climates in these areas compared to present times [56,199]. In the Late Holocene, the species was also present on the Chilean side of Isla Grande de Tierra del Fuego [200] and is the only known mustelid that reached this island. Nonetheless, as aridity decreased and the climate became more humid, the geographic range of *Lyncodon* diminished, which suggests that during the cold, dry, and arid periods of South America during the Miocene and Pliocene, the ancestors of this genus were able to thrive.

Considering that a good part of the analyses carried out (genetic distances for the mt*NADH5* and *Dloop* markers, DT trees, and the three MJ networks) show a tendency towards a greater relationship between the sequences of *L. patagonicus* with *G. cuja* than of the latter with *G. vittata*, our hypothesis maintains that the next ancestor to appear after that of *L. patagonicus* was that of *G. cuja*. As the climate became more humid and semi-desert and steppe zones receded, an ancestor of the branch that gave rise to *L. patagonicus* began to colonize more northerly zones, dry and partially humid forests, and steppes at varying altitudes. This coincided with an increase in prey variety, leading to a corresponding increase in size and resulting in the somewhat larger body of present-day *G. cuja*. This species occurs in multiple habitats from sea level to 4200 masl. It may be abundant in open habitats [23,35]. Therefore, this grison colonized more diverse environments (with a greater variety of prey) than the direct ancestor of *L. patagonicus*, resulting in a selective process that favored increased body size due to the availability of more diverse and larger prey. Some authors have postulated that different climatic conditions affect the body and cranial size of different populations of *G. cuja* living in different environments [7]. In fact, Miglioni et al. [7] indicated the significant association between skull size and latitude, following the predictions of Bergmann’s rule [201]. This rule establishes that within endothermic species, specimens from colder climates or higher latitudes are generally larger than those from warmer regions or lower latitudes. Notwithstanding, we do not believe that Bermann’s rule, nor directly the climatological conditions, are what directed the evolution of body size in Neotropical Lyncodontini from southern South America to northern South America, as we will discuss below.

Finally, during its northward colonization, this lineage penetrated humid forests where the variety, quantity, and size of prey increased, as did the number of competing predators. Consequently, its size increased, giving rise to the present-day *G. vittata*. This view aligns with the assertion by Koepfli et al. [10] that mustelids display such substantial ecomorphological diversity within one family that they provide a striking example of the rapid diversification of species into different ecological niches from a common ancestor (adaptive radiation). In the case of *G. vittata*, it is a species often found near rivers, streams, and wetlands, from sea level to 1500 masl, but mostly below 500 masl [26]. In some countries, *G. vittata* and *G. cuja* coincide, but they do so in different biomes or ecoregions [202]. For example, *G. vittata* in Peru seems to occur in the broad region of the Peruvian Amazon but not in the Peruvian Andes or the drier region of the Pacific coast. In contrast, *G. cuja* inhabits the southern Peruvian Andes in the montane grassland and scrublands (Puna grasslands) region. In Bolivia, the only record of *G. vittata* originates from the Tropical and Subtropical Dry Broadleaf Forest (Chiquitano), whereas those for *G. cuja* include this same biome as well as other Bolivian Andean regions. In Paraguay, both species seem to co-exist in the Tropical and Subtropical Moist Broadleaf Forests (Atlantic Forest) in the southern region of the country, although *G. cuja* also occurs in the Savanna (Chaco). Within Brazil, there seems to exist a clear biogeographical division between the species, with *G. vittata* exclusively present in the Amazon basin and *G. cuja* occurring in other biomes, including the drier Cerrado (savanna) and Caatinga (deserts and xeric shrublands) of the northeast, as well as the Atlantic Forest throughout the eastern seaboard and the pampas grasslands towards southern Brazil. Therefore, *G. vittata* is much more actively linked to humid forest environments than the other two Neotropical Ictonychinae species. Bonrnholdt et al. [5] examined a total of 67 specimens of *G. vittata*, whose identification was confirmed based on morphological characteristics, and they did not find evidence corroborating the occurrence of *G. vittata* in northeastern, southeastern, or southern Brazil. Likewise, these authors observed that the northernmost limit of *G. vittata* is the Mexican provinces of San Luis Potosi and Veracruz. This region is the boundary between humid and semi-humid forests in southern Mexico to drier and more open regions in northern Mexico (where *G. vittata* seems not to be found). This is an interesting distributional pattern because it is very similar to that observed in the southernmost limits of the species.

When two morphologically and/or ecologically similar species geographically overlap, a change in size or morphology is expected to minimize competition. Both species may have competed for resources at the boundary of their ranges, which would inhibit pervasive geographical overlap [203]. This could have occurred when the lineage that gave rise to *C. cuja* began to diverge into the lineage that would expand further north, becoming *G. vittata*. It is possible that the earlier geographical distribution of both species overlapped more than it does today. The fossil records of both species already occurred in Lagoa Santa in the Brazilian Cerrado biome [204]. Likewise, there are fossil remains of *G. cuja* in Pleistocene deposits in the northern Cerrado, at Aurora, in the state of Tocantins, bordering the Amazon. These data show that the extant southern South American distribution of *G. cuja* and the northern South American distribution of *G. vittata* may have been more sympatric than they are today and that their contact area may have varied more recently due to climate change [204].

Another hypothesis that could be key to understanding the body size variation from south to north in the Neotropics is that proposed by McNab [205], known as the “resource rule.” This rule states that spatial variation in mammal body size can be explained by the availability and characteristics of the resources consumed. Mustelids have higher energy needs than expected for their body mass, which may require a larger prey size to satisfy their energetic requirements [205]. For example, *G. cuja* feeds on rabbits in Argentina and is significantly larger than specimens from southern Brazil, which feed exclusively on small rodents, despite the high abundance of *Lepus europaeus* in the region [206], where there is no evidence of its consumption. The number and size of species are greater in the tropical areas inhabited by *G. vittata* than in the regions inhabited by *G. cuja*, which could have selected for a larger body size, as could the presence of the metaconid in the lower carnassial, which might help in chewing larger pieces of prey. Notwithstanding, this hypothesis can be combined with another, according to which the size difference among the three extant Neotropical Lyncodontini species is mostly influenced by trophic competition with other living or extinct mustelids and not directly among themselves. Among the extinct taxa that might have driven such size evolution are other species of the genus *Galictis*, whose presence is well-known in the South American fossil record [171,189]. The concurrent existence of these congeneric species throughout the late Pliocene and Pleistocene may support the hypothesis that they played a competitive role in shaping the size of the extant Neotropical Lyncodontini species, just as other competing carnivores such as other Mustelidae (*E. barbara*, for instance), Felidae, and Canidae may have done [207]. Additionally, *G. cuja* occupies savannas, steppes, rocky areas, and forests, and its adaptation to different habitats might have led to its observed variation in coat color, which is greater than that found in *G. vittata*. Furthermore, this species often reaches high altitudes and latitudes, which may have historically led to selective pressures for denser fur. The situation of *G. vittata* is different, as it exhibits a more homogeneous coloration. The mixture of black and white fur, producing a pale gray coat, might be favored in dense vegetation and darker landscapes, such as tropical forests, where *G. vittata* lives. This would allow it to remain less conspicuous to its prey and avoid larger and more competitive predators. Additionally, the shorter and sparser fur in this species is likely an adaptation to the warm temperatures prevalent throughout its geographical range [5]. Therefore, our knowledge of the biogeographical distribution and morphological evolution of Neotropical Lyncodontini can be reconciled with the molecular results obtained to propose the hypothesis of a southern-to-northern South American evolution of present-day Neotropical Lyncodontini species.

(3) Although there are few genetic population studies with Neotropical Lyncodontini, they can provide some data favorable to the hypothesis presented here. When we compare the genetic diversity values (no data exist for *L. patagonicus*) between *G. cuja* and *G. vittata*, we observe that the H_d_ values for *G. cuja* in different areas of Brazil are higher [H_d_ = 0.92 in southern (Rio Grande do Sul, Santa Catarina, and Paraná states) and southeastern (Federal District and Brazilian states of São Paulo and Minas Gerais) areas of Brazil [5] and H_d_ = 0.90 in the states of Paraíba, Minas Gerais, and Rio de Janeiro [6]] than those found in the present study for *G. vittata*, including Guatemala, Colombia, Ecuador, Peru, Bolivia, and French Guiana (H_d_ = 0.66). The π values were similar in the three studies (around 1%), but the geographic area considered for *G. vittata* was significantly larger, including more biomes, so the π value can be considered proportionally lower in *G. vittata* than in *G. cuja*. This difference in genetic diversity could be explained by the central–marginal hypothesis [208,209]: the central distribution range of a species tends to exhibit higher levels of genetic diversity than the populations located at the periphery of the distribution range. This could indicate that *G. cuja* acts more like a central population and *G. vittata* more like a peripheral population derived from the former. Similarly, nucleotide and haplotype diversity within these populations from Brazil are different [5]. Southern Brazil has high values of H_d_ (0.87 ± 0.03) and moderate values of π (0.99 ± 0.49 in %), while southeastern Brazil has higher H_d_ (0.92 ± 0.09) but lower π (0.28 ± 0.18 in %). Similarly, the southern Brazilian population historically showed a stable population, while the southeastern Brazilian population showed signs of population expansion [5]. Although no solid genetic structure exists, the split between the two major *G. cuja* groups in Brazil (considering the concatenated data of mt*NADH5* and mt*Dloop*) implies at least 15 mutational steps, which is considerably deep, especially considering that it is not geographically localized [16]. This suggests that the *G. cuja* population in southern Brazil is more original and older than that of southeastern Brazil or more northerly areas in central and coastal Brazil, which shows signs of more recent expansion. This aligns well with a south-to-north colonization pattern like the one we propose in this work. Similarly, Bontempo et al. [6] found that genetic diversity based on the mt*Cyt-b* gene identified high haplotypic diversity, although with low nucleotide diversity, suggesting that this population underwent population expansion and confirming the presence of gene flow. Filippini [79] obtained the same results for the Argentinian and Brazilian data taken together. *G. vittata* also showed signs of population expansion, at least for the marker in which the most samples were studied (mt*NADH5*), which could be related to its more recent existence. In turn, the mt*NADH5* gene haplotypes of *G. cuja* from Argentina (even further south) that we studied appear to have given rise to the haplotypes of southern Brazil (south–north colonization, again). On the other hand, the Argentinian specimens studied, even though they had more original haplotypes, were basically genetically impoverished, exhibiting genetic diversities close to 0 [16,79]. Even though the Argentinian population is more ancestral than the Brazilian ones, it may have more recently experienced a bottleneck or may have become extinct, reconstituting itself from very recently acquired populations in southern Brazil that possessed original haplotypes. Another very interesting genetic data point that reinforces our hypothesis of south–north colonization and speciation for Neotropical Lyncodontini is that presented by Bornholdt et al. [5]. These authors showed a haplotype network with the nuclear *RAG1* gene, where the position of the root was based on two outgroup species (*Poecilogale albinucha* and *Ictonyx striatus*). The *G. vittata* haplotype was derived from a central haplotype of *G. cuja*, another genetic indication of south–north colonization and speciation in the Neotropical Lyncodontini.

Of all of the genetic data obtained, only one could introduce doubt regarding the hypothesis we present. The two specimens from Guatemala and northern Colombia (putatively *G. v. canaster*) showed in different phylogenetic trees (especially DT trees of the mt*NADH5* and mt*12S rRNA* genes and in some MJ networks) that they originated earlier than the remaining South American haplotypes for *G. vittata*. This could be consistent with north–south colonization for this species. An alternative hypothesis is that the *L. patagonicus* and *G. cuja* lineage (but this would require admitting that the genus *Galictis* is paraphyletic) arrived in South America first, taking advantage of the early development of prominent Northern Hemisphere glaciations [183]. This supports the hypothesis of Webb [210], who proposed that proto-GABI dispersals were aided by the development of Northern Hemisphere glaciations. These promoted the development of savanna-like ecologies in Central America, in contrast to their generally tropical character, and thereby permitted savanna-adapted mammals to cross between North and South America. Already in South America, the ancestor of *L. patagonicus* would have given rise to the ancestor of *G. cuja*. Meanwhile, in the tropical and forested part of Central America, the ancestor of *G. vittata* would have arisen, which more recently penetrated the tropical part of north–central South America. The problem with this alternative hypothesis lies in two points. The first is that not all molecular analyses show the paraphyly of the genus *Galictis*, and the second is that all of these processes would have had to occur in the last approximately 2.8 mya, and all our estimates of temporal divergence, in general, exceed those dates. It would be very important to be able to determine the nonsense and frameshift mutations in *TAS1R1* and *TAS1R3* genes in *G. vittata*. Depending on the results obtained, it would be possible to support the colonization of Lyncodintini from north to south or from south to north of South America (or the possibility that *L. patagonicus* and *G. cuja* are more strongly associated with each other, while *G. vittata* forms another distinct lineage, and the taxonomic and systematic implications this could have).

The proposed hypotheses can only be considered if much larger genetic datasets can be generated. Here, we have presented a first outline with a very limited number of specimens and genetic markers, intended merely as a small seed for a possible reinterpretation of the colonization and speciation of important South American mammal species. This requires studying a much larger number of specimens for the three Neotropical Lyncodintini species, especially *L. patagonicus* and *G. vittata*. Furthermore, the analysis of complete mitogenomes and, if possible, whole metagenomes that effectively represent these three species is necessary.

## 5. Conclusions

The main conclusions of this study are as follows. (1) The use of small fragments of three mt markers (*NADH5*, *12S rRNA*, and *D-loop*) proved to be very useful for recovering relevant information on the evolutionary history of Neotropical Lyncodontini species of the genera *Galictis* and *Lyncodon* from very small samples from which only very small amounts of DNA of lesser quality could be extracted. (2) This study used the largest population sample analyzed to date for *G. vittata* (13 specimens from Guatemala, Colombia, Ecuador, Peru, Bolivia, and French Guiana). Previously, only three individuals presumed to be of Peruvian origin had been studied [5]. Similarly, for the first time, mitochondrial sequences for the three previously mentioned markers were obtained from one or two specimens of *L. patagonicus* (to date, only a sequence of the mt*Cyt-b* gene [12] had been generated for this species). (3) The nucleotide diversity values obtained for each of these three markers in *G. vittata* corresponded to the evolution rate per million years for each of the three markers. The mt*Dloop* marker (evolution rate per million years of 4%) showed the highest nucleotide diversity value (2.1%), followed by the mt*NADH5* gene (1.46%) (evolution rate per million years of 1.22%), and, finally, the mt*12S rRNA* gene showed the lowest nucleotide diversity (0.145%), also being the marker with the lowest evolution rate per million years (0.42%). (4) The haplotype and nucleotide diversity of *G. vittata* was lower than the diversities measured in other studies for *G. cuja* in southern and southeastern Brazil [6,12], even though *G. vittata* was sampled over a wider geographic range and included more biomes. (5) For the mt*NADH5* gene, evidence from two analyses revealed possible population expansion of the female lineages. Using the mismatch distribution procedure, it was estimated that the origin of this population expansion could have begun between 635,000 and 370,000 ya. The BSP procedure with the mt*NADH5* gene detected slow but continuous population growth from 500,000 to 250,000 ya. From that point, population growth intensified until 80,000 ya. Population contraction was detected around 40,000 ya. This population decrease continued until it reached its smallest size at approximately 10,000 ya. From about 10,000 ya (Holocene) to about 1000 ya, the population of this species grew again, only to decline somewhat in recent centuries. (6) Genetic heterogeneity analyses, BAPS analyses, and phylogenetic trees using the mt*NADH5* gene detected three genetically distinct groups within *G. vittata*. One group was in Central America and northern Colombia, another in French Guiana, and the third in the rest of South America. Analyses using the mt*12S rRNA* gene also detected three distinct groups in some cases, but, in other analyses, they detected only two distinct populations: the Central American/northern Colombian group and the group from the rest of South America. The taxonomic nomenclature each of these groups should receive was discussed. (7) The small mt*DNA* fragments used showed phylogenetics results basically identical to those found in two previous studies using sequences of multiple nuclear genes and the mt*Cyt-b* gene, which highlights the important contributions that can be made from an evolutionary perspective even with small amounts of low-quality DNA from species that are difficult to sample. (8) Phylogenetic trees and MJ networks showed in all cases that the *E. barbara* lineage was the oldest of all of the mustelid genera studied. (9) The divergence times of the ancestor of *E. barbara* and of the ancestors of the Lutrinae and Mustelinae were practically identical to those obtained by Koepfli et al. [10] and relatively like those obtained by Sato et al. [12]. (10) Nevertheless, the origin of Neotropical Lyncodontini was older than in other studies (late Miocene, early Pliocene). (11) Some of the analyses detected a closer relationship between *L. patagonicus* and *G. cuja* than the latter with *G. vittata*. This could show that *Galictis* might be a paraphyletic genus and would have an impact on the nomenclature of those genera. (12) Furthermore, all of the DT trees and MJ networks detected that the *L. patagonicus* haplotypes were the first to originate, followed by those of *G. cuja*, with *G. vittata* showing the most recent haplotypes. This would indicate that the origin of the current Neotropical Lyncodontini occurred in the south of the continent and that colonization towards northern South America began from there. Nonetheless, other alternative hypotheses cannot be ruled out.

## Figures and Tables

**Table 1:** Geographical sources and types of tissue of the mustelids collected and analyzed at three mitochondrial markers (*NADH5, 12S rRNA*, *Dloop*). IAvH-M = Museum of Institute Alexander von Humboldt with the code of the specimens.

Species and Locations	Number of Specimens Analyzed	Types of Tissues Employed to Obtain DNA
***Galictis vittata***	13	
**Guatemala:**		
Jalapa department	1 (obtained from nature)	hairs
**Colombia:**		
Colosó, Sucre department	1 (obtained from IAvH-M-5547)	skin
Meta department	1 (obtained from IAvH-M-5381)	skin
**Ecuador:**		
Puyo, Pastaza province	1 (obtained from nature)	hairs
Fatima, Macas, Morona-Santigo province	1 (obtained from nature)	hairs
**Peru:**		
Quistococha, Loreto department	3 (obtained from nature)	hairs
Yarinacocha, Ucayali department	1 (obtained from nature)	hairs
**Bolivia:**		
Chimoré River, Cochabamba department	1 (obtained from nature)	fragment of mandible with teeth
**French Guiana:**		
Roura, Cayenne district	1 (obtained from nature)	hairs
Régina, Saint-Georges district	1 (obtained from nature)	hairs
Camopi, Saint-Georges district	1 (obtained from nature)	hairs
***Galictis cuja***	2	
**Argentina:**		
Sierra Ventana, Partido Tornquist, Buenos aires province	1 (obtained from nature)	skin
La Plata, Buenos Aires province	1 (obtained from nature)	hairs
***Lyncodon patagonicus***	2	
**Argentina:**		
Yuquiche, Río Negro province, Patagonia	1 (obtained from nature)	skin
Salinas Grande, Malachín, La Pampa province	1 (obtained from nature)	skin
***Eira barbara***	2	
**French Guiana:**		
Savane du Petit, Cayenne district	1 (obtained from nature)	hairs
Petit Saut-Sinnamary, Cayenne district	1 (obtained from nature)	hairs
***Neogale frenata***	2	
**Colombia:**		
La Calera, Cundinamarca department	1 (obtained from nature)	skin
**Ecuador:**		
Píllaro, Tungurahua province	1 (obtained from nature)	skin
***Lontra longicaudis***	2	
**Peru:**		
Itaya River, Loreto department	1 (obtained from nature)	skin
Nanay River, Loreto department	1 (obtained from nature)	skin
***Lontra provocax***	1	
**Chile:**		
Quilo, Chiloe Island	1 (obtained from nature)	skin
***Lontra felina***	1	
**Chile:**		
Playa Brava, Iquique, Tarapacá	1 (obtained from nature)	skin

**Table 2:** Mitochondrial (mt) genetic diversity at three mt markers (*NADH5*, *12S rRNA*, *Dloop*) analyzed in the mustelid *Galictis vittata*. H = number of haplotypes; H_d_ = haplotypic diversity; π = nucleotide diversity; θ_per sequence_ (=N_e_µ); SD = standard deviation.

mt Genes	H	H_d_ ± SD	π ± SD in %	θ_per sequence_
***NADH5***	8	0.88 ± 0.07	1.46 ± 0.29	4.83 ± 2.15
***12s rRNA***	3	0.35 ± 0.17	0.148 ± 0.08	0.68 ± 0.52
***D-Loop***	3	0.83 ± 0.22	2.08 ± 0.5	3.82 ± 2.38

**Table 3:** Overall genetic heterogeneity among three different *Galictis vittata* populations detected in Central and South America at the mitochondrial *NADH5* gene. * *p* < 0.05 (significant), ** *p* < 0.001 (highly significant); df = degree of freedom; Nm = gene flow.

Genetic Heterogeneity and Gene Flow Statistics	Values	Probabilities
χ^2^	26.00 df = 14	0.026 *
H_ST_	0.24	0.0012 *
K_ST_	0.57	0.00001 **
K_ST_ *	0.51	0.00001 **
Z_S_	14.71	0.00001 **
Z_S_ *	2.54	0.00001 **
S_nn_	0.96	0.00001 **
γ_ST_	0.67	0.0004 **
N_ST_	0.67	0.0005 **
F_ST_	0.66	0.0005 **
Nm (γ_ST_)	0.24	
Nm (N_ST_)	0.25	
Nm (F_ST_)	0.25	

**Table 4:** Genetic heterogeneity statistics [F_ST_ and γ_ST_ (into parenthesis)] among *Galictis vittata* populations at the mitochondrial *NADH5* gene below the diagonal; Kimura 2P genetic distances (in %) among *Galictis vittata* populations at the mitochondrial *NADH5* gene above the diagonal. Group 1: Central America and northern Colombia; Group 2: French Guiana; Group 3: the rest of South America. * *p* < 0.05 (significant), ** *p* < 0.001 (highly significant).

Populations	Group 1	Group 2	Group 3
Group 1	-	2.4 ± 0.9	1.3 ± 0.7
Group 2	0.71 **	-	1.3 ± 0.7
	(0.78) **		
Group 3	0.51 *	0.82 **	-
	(0.50) *	(0.60) **	

**Table 5:** Overall genetic heterogeneity between two different *Galictis vittata* populations detected in Central and South America at the mitochondrial *12S rRNA* gene. * *p* < 0.05 (significant), ** *p* < 0.001 (highly significant); df = degree of freedom; Nm = gene flow.

Genetic Heterogeneity and Gene Flow Statistics	Values	Probabilities
χ^2^	11.00 df = 2	0.0041 *
H_ST_	1.00	0.0164 *
K_ST_	0.64	0.0166 *
K_ST_ *	1.00	0.0164 *
Z_S_	17.50	0.0158 *
Z_S_ *	2.92	0.0164 *
S_nn_	0.92	0.0166 *
γ_ST_	0.80	0.0002 **
N_ST_	0.67	0.0004 **
F_ST_	0.67	0.0004 **
Nm (γ_ST_)	0.12	
Nm (N_ST_)	0.25	
Nm (F_ST_)	0.25	

**Table 6:** Demographic change statistics applied to three mitochondrial markers (*NADH5, 12S rRNA, Dloop*) for *Galictis vittata*. *p* = probability. * Significant.

Statistics	*NADH5*	*12S rRNA*	*D-Loop*
Tajima’s D	−0.83 (*p* = 0.21)	−0.78 (*p* = 0.21)	0.47 (*p* = 0.73)
Fu’s Fs	−1.44 (*p* = 0.19)	−0.66 (*p* = 0.14)	1.16 (*p* = 0.69)
Fu & Li’s D *	−0.51 (*p* = 0.31)	−0.33 (*p* = 0.23)	0.47 (*p* = 0.75)
Fu & Li’s F *	−0.62 (*p* = 0.27)	−0.45 (*p* = 0.26)	0.47 (*p* = 0.74)
R_2_	0.10 (*p* = 0.046) *	0.16 (*p* = 0.26)	0.20 (*p* = 0.16)
Raggedness (*rg*)	0.02 (*p* = 0.005) *	0.25 (*p* = 0.53)	0.75 (*p* = 0.90)

**Table 7:** Kimura 2P genetic distance (in %) among the different mustelid species analyzed in this study at the mitochondrial *NADH5*, *12S rRNA*, and *Dloop* markers. Genetic distance values of mitochondrial *NADH5* and *12S rRNA* (into parenthesis) genes below the diagonal; genetic distance values of mitochondrial *Dloop* marker above the diagonal.

Species	*Galictis vittata*	*Galictis cuja*	*Lyncodon patagonicus*	*Neogale frenata*	*Lontra longicaudis*	*Lontra provocax*	*Lontra felina*	*Eira Barbara*
*Galictis vittata*	-	6.3 ± 1.9	10.3 ± 2.7	15.4 ± 3.5	21.3 ± 5.1	-	18.9 ± 4.1	13.3 ± 3.4
*Galictis cuja*	18.7 ± 3.5	-	7.3 ± 2.0	13.7 ± 3.2	21.3 ± 5.1	-	17.0 ± 3.7	13.4 ± 3.4
	(1.8 ± 0.7)							
*Lyncodon patagonicus*	17.7 ± 3.7	16.0 ± 3.6	-	10.1 ± 2.9	16.0 ± 4.0	-	11.4 ± 2.7	7.1 ± 2.3
	(1.8 ± 0.8)	(2.4 ± 1.0)						
*Neogale frenata*	23.5 ± 4.8	22.7 ± 4.1	22.9 ± 4.9	-	14.4 ± 3.7	-	5.9 ± 2.0	11.8 ± 3.4
	(5.8 ± 1.3)	(5.8 ± 1.4)	(4.4 ± 1.1)					
*Lontra longicaudis*	25.4 ± 4.6	26.6 ± 5.2	22.0 ± 4.4	19.7 ± 3.7	-	-	11.4 ± 3.1	17.1 ± 4.4
	(6.1 ± 1.5)	(5.7 ± 1.6)	(5.4 ± 1.5)	(4.4 ± 1.4)				
*Lontra provocax*	25.8 ± 4.8	27.9 ± 5.7	24.0 ± 5.0	19.5 ± 3.7	5.0 ± 1.8	-	-	-
	-	-	-	-	-			
*Lontra felina*	-	-	-	-	-	-	-	16.0 ± 4.0
	-	-	-	-	-	-		
*Eira barbara*	23.8 ± 4.6	33.6 ± 6.9	25.5 ± 5.1	21.9 ± 4.4	23.3 ± 4.6	25.2 ± 5.1	-	-
	(7.2 ± 1.5)	(7.7 ± 1.7)	(5.7 ± 1.1)	(6.5 ± 1.5)	(6.4 ± 1.5)	-	-	

**Table 8:** Temporal separations between different mustelid taxa estimated using Bayesian inference (BI) and NJ networks (±standard deviation) with three mitochondrial markers (*NADH5*, *12S rRNA*, *D-loop*). Confidence intervals, HPD, at 95% are shown in parentheses for BI. Times are in millions of years. Some estimations are compared with those obtained by Koepfli et al. [10] and Sato et al. [12].

Relationships Between Taxa	*NADH5*	*12S rRNA*	*D-loop*	Koepfli et al. [10]	Sato et al. [12]
Split between ancestor of *Eira Barbara* and the rest of the Mustelidae studied	BI: 11.34 (9.38–13.37)	BI: 11.34 (9.41–13.28)	BI: 11.75 (10.47–13.10)	11.1 (9.2–12.9)	
Split between Ictonychinae and Mustelinae–Lutrinae	BI: 10.41 (7.98–12.66)	BI: 9.47 (6.53–12.28)	BI: 9.81 (8.28–11.36)	9.3 (7.6–10.9)	Split between Ictonychinae and Lutrinae: 9.80 (8.44–11.24)
					10.24 (8.63–12.11)
Split between Mustelinae and Lutrinae	BI: 8.66 (5.96–11.39)	BI: 6.49 (3.75–9.57)	BI: 7.88 (6.23–9.54)	8.8 (7.3–10.4)	10.49 (9.03–12.01)
	NJ: 6.1 ± 0.88	NJ: 6.42 ± 1.30	NJ: 6.03 ± 0.41		10.60 (8.99–12.48)
Split between *Lontra provocax* and *Lontra longicaudis*	BI: 1.84 (0.88–3.08)				
	NJ: 1.86 ± 0.38				
Split between *Lontra felina* and *Lontra longicaudis*			BI: 3.27 (0.51–6.91)	1.8 (0.6–3.2)	
			NJ: 1.18 ± 0.26		
Split between *Lyncodon patagonicus*–*Galictis cuja* and *Galictis vittata*	BI: 6.88 (4.76–9.23)				
Split between *Lyncodon patagonicus* and *Galictis cuja-Galictis vittata*		BI: 5.00 (2.73–7.63)	BI: 5.13 (1.73–8.87)		2.61 (2.42–2.88)
					2.89 (2.42–3.76)
Split between *Galictis cuja* and *Galictis vittata*	NJ: 4.23 ± 0.11	BI: 3.43 (1.77–5.44)	BI: 2.25 (0.61–4.66)	2.8 (1.1–5.1)	1.67 (1.17–2.17)
		NJ: 1.38 ± 0.50	NJ: 1.85 ± 1.23		2.03 (1.34–2.86)
Initial diversification within *Galictis cuja*	BI: 0.85 (0.36–1.5)				
Split between Central American and northern Colombian *Galictis vittata* and the rest of South American *Galictis vittata*	BI: 1.32 (0.69–2.12)	BI: 1.5 (0.6–2.7)			
	NJ: 0.67 ± 0.23	NJ: 0.43 ± 0.26			
Split between French Guianan *Galictis vittata* and the rest of South American *Galictis vittata*	BI: 0.82 (0.39–1.64)				

## Data Availability

The datasets generated and analyzed during the current study are available from the corresponding author upon reasonable request at the e-mails mruizgar@yahoo.es and mruiz@javeriana.edu.co. The GenBank accession numbers are as follows: in processing to submit to GenBank.
